# Treatment options for cisplatin-ineligible patients with locally advanced head and neck squamous cell carcinoma: a systematic review

**DOI:** 10.1007/s00432-024-05887-z

**Published:** 2024-08-02

**Authors:** Isabella Michelon, Gilca Costa Nachtigal, Maria Inez Dacoregio, Ana Cristina Beitia Kraemer Moraes, Mauricio Moraes, Lívia Silva Piva, Catiara Terra da Costa, Rafael Guerra Lund, Douver Michelon

**Affiliations:** 1 Department of Medicine, Catholic University of Pelotas, Pelotas, Brazil; 2 Department of Internal Medicine, Federal University of Pelotas Teaching Hospital (EBSERH), Pelotas, Brazil; 3 Department of Medicine, University of Centro Oeste, Guarapuava, Brazil; 4 Department of Surgery, Faculty of Medicine, Catholic University of Pelotas, Pelotas, Brazil; 5 Graduate Program in Dentistry, School of Dentistry, Federal University of Pelotas, Pelotas, RS 96015560 Brazil; 6 Department of Medicine, Federal University of Pelotas, Pelotas, Brazil

**Keywords:** Head and neck squamous cell carcinoma, HNSCC, Cisplatin, Ineligible, Chemotherapy

## Abstract

**Purpose:**

There is no agreed-upon standard option for patients with locally advanced head and neck squamous cell carcinoma (LA HNSCC) unfit for cisplatin-based regimens. Therefore, we performed a systematic review to explore alternative options for this population.

**Methods:**

We searched PubMed, Cochrane, and Embase databases for observational studies and clinical trials (CTs) assessing treatment options for LA HNSCC cisplatin-ineligible patients. This study was registered in PROSPERO under the number CRD42023483156.

**Results:**

This systematic review included 24 studies (18 observational studies and 6 CTs), comprising 4450 LA HNSCC cisplatin-ineligible patients. Most patients were treated with cetuximab-radiotherapy [RT] (50.3%), followed by carboplatin-RT (31.7%). In seven studies reporting median overall survival (OS) in patients treated with cetuximab-RT, it ranged from 12.8 to 46 months. The median OS was superior to 40 months in two studies assessing carboplatin-RT, and superior to 15 months in two studies assessing RT alone. For other regimens such as nimotuzumab-RT, docetaxel-RT, and carboplatin-RT plus paclitaxel the median OS was 21, 25.5, and 28 months, respectively.

**Conclusions:**

Our systematic review supports the use of a variety of therapy combinations for LA HNSCC cisplatin-ineligible patients. We highlight the urgent need for clinical studies assessing treatment approaches in this population.

**Supplementary Information:**

The online version contains supplementary material available at 10.1007/s00432-024-05887-z.

## Introduction

Head and neck cancer (HNC) is among the ten most common cancers globally (Barsouk et al. 2023). Each year about 4.5% of all cancer diagnoses and deaths are accounted for HNC (Barsouk et al. 2023). Roughly 90% of HNC are squamous cell carcinomas (SCC) and a great percentage of patients are diagnosed with locally advanced disease (Dhull et al. 2018; Haddad et al. 2023).

The mainstay of treatment for locally advanced head and neck squamous cell carcinoma (LA HNSCC) is surgery followed by adjuvant chemoradiotherapy (CRT) or CRT alone (Machiels et al. 2020; Pfister et al. 2020). For unresectable tumors or patients with poor functional status and contraindications to surgery, CRT is the recommended option (Machiels et al. 2020; Pfister et al. 2020). Regardless of tumor location, concomitant CRT with high-dose cisplatin (> 200 mg/m^3^) has been proved superior to RT alone (Machiels et al. 2020; Porceddu et al. 2020). The benefit is consistent whether using standard or altered fractionation RT regimens (Machiels et al. 2020). Among cisplatin schedules, the 3-weekly regimen is the preferred option.

Nevertheless, CRT accounts for important treatment-related morbidity. Among the several complications of RT, swallowing impairment and aspiration in HNC patients notably impact overall survival (De Ruysscher et al. 2019; Trotti et al. 2003). As to cisplatin, the most common and debilitating adverse events include gastrointestinal symptoms, nephrotoxicity, and peripheral neuropathy (Barabas et al. 2008). Accordingly, the study by Espeli et al. estimated that about 50% of patients on high-dose cisplatin were unable to complete the planned treatment schedule due to its toxicity (Espeli et al. 2012).

Given the complexity of HNSCC clinicopathological features, a multidisciplinary approach is essential (De Felice et al. 2019). To optimize clinical decisions, multidisciplinary programs assess all individual features implicated in treatment strategies (Ang 2008; De Felice et al. 2019). Additionally, these programs cover a variety of supportive measures and services that are crucial in maintaining quality of life and treatment adherence, including nutritional assessment, pain management, and rehabilitation (Ang 2008; De Felice et al. 2019). This is of special relevance in patients unable to tolerate cisplatin-based treatments and who often present with a high burden of frailty and comorbidities.

A retrospective study conducted in a cohort of veterans in the United States estimated that about one-third of HNSCC patients were classified as ineligible for cisplatin and were treated with alternative regimens (Sun et al. 2022). Yet, international guidelines do not support specific regimens due to the scarce evidence for HNSCC cisplatin-unfit patients (Koyfman et al. 2019; Machiels et al. 2020). A combination of docetaxel (DTX), cetuximab (CTX), and RT is outlined in the National Comprehensive Cancer Network (NCCN) guidelines as alternative agents for this population (NCCN Clinical Practice Guidelines in Oncology. Head and Neck Cancer., n.d.). Some authors support the use of carboplatin (CB)-based regimens, 5-fluorouracil, and RT alone as feasible options (Haddad et al. 2023; Sun et al. 2022). In contrast, studies have shown that CTX-RT is superior to RT alone, whereas retrospective data support CB-based regimens over CTX (Bonner et al. 2006; Sun et al. 2022).

Other regimens currently being assessed include DTX plus RT and the combination of immune checkpoint inhibitors and RT (Patil et al. 2023; Tao et al. 2023). However, there is a lack of consensus concerning the optimal agent for this population. In light of heterogeneous and limited evidence for this group of patients, we performed a systematic review to compile all available information on treatment options for cisplatin-ineligible patients with LA HNSCC within clinical trials and in the real-world setting.

## Materials and methods

This study was performed and reported according to the guidelines from the Cochrane Collaboration and the Preferred Reporting Items for Systematic Reviews and Meta-Analysis (PRISMA) (Page et al. 2021). It was registered in the International Prospective Register of Systematic Reviews (PROSPERO [CRD42023483156]). Initially, this study was planned as a systematic review and meta-analysis. However, due to important differences in the design of studies and outcomes, only a systematic review was performed. The PRISMA checklist for the abstract and the manuscript is available in Supplementary Table S1.

### Data source and search strategy

The initial systematic search was conducted on PubMed, Embase, and Cochrane databases on September 20th, 2023, and it was last updated on November 16, 2023. The following combination of medical subject headings (MeSH) terms and boolean connectors were used: “head and neck” AND “cisplatin” AND “ineligible”. The full search strategy used on each database is available for reference in Supplementary Table S2.

### Eligibility criteria

The main eligibility criteria consisted of published phase II or III clinical trials (CTs) and prospective or retrospective cohort studies evaluating treatment options in patients with LA HNSCC and contraindications for cisplatin. We included studies on squamous cell cancers mainly located in the oral cavity, oropharynx, hypopharynx, and larynx. To be eligible for inclusion, studies were required to report at least one survival or response outcome as listed below. No specific restrictions were made as to the publication date, presence of a control/comparator group, number of patients, or the treatment regimen assessed.

The main exclusion criteria were as follows: (1) unclear or no information if patients were cisplatin-ineligible; (2) metastatic/recurrent HNSCC patients; (3) cancers located in the brain, eye, esophagus, thyroid or skin of the head and neck or non-squamous cell cancers; (4) patients on induction chemotherapy regimens; (5) only safety data reported; (6) abstracts from conferences and not original studies; and (8) patients who switched from standard cisplatin regimens to low-dose cisplatin. Due to differences in outcomes and prognosis, studies analyzing exclusively squamous cell nasopharyngeal carcinomas or SCC of paranasal sinuses were excluded.

### Data collection and outcomes

Three authors (IM, GCN, MID) independently screened the studies by title and abstract, selected the articles for full-text review, and extracted data from included studies. Other authors (DM and RGL) were consulted in case of inconsistencies. Data was collected from individual studies on the study design, study location, number of patients, and patients' baseline characteristics (*e.g.*, sex, age, tumor stage).

Our primary outcome of interest was overall survival (OS). Secondary outcomes include (1) progression-free survival (PFS); (2) locoregional control (LRC); (3) disease-free survival (DFS); (4) incidence of distant metastases or recurrence; (5) locoregional failure (LRF); (6) objective response rate (ORR); (7) disease control rate (DCR); and (8) adverse events (AEs). All outcomes were presented in tables according to the information available in individual studies.

Considering that some outcome definitions and treatment schedules varied across studies, supplemental tables with the eligibility criteria and treatment details of each included study (Supplementary Table S3) and outcome definitions (Supplementary Table S4) are available for reference. Other responses and survival outcomes not included in main tables are described in Supplementary Table S5. Details about the included population are presented in Supplementary Table S6.

### Quality assessment

Three authors (IM, GCN, and MID) independently conducted the risk of bias assessment. Inconsistencies were resolved by consensus or by consulting other authors (DM and RGL). The risk of bias in non-randomized studies was explored using ROBINS-I, and for randomized studies, RoB 2 tool was used (Sterne et al. 2016, 2019).

## Results

### Baseline characteristics

**Fig. 1:**
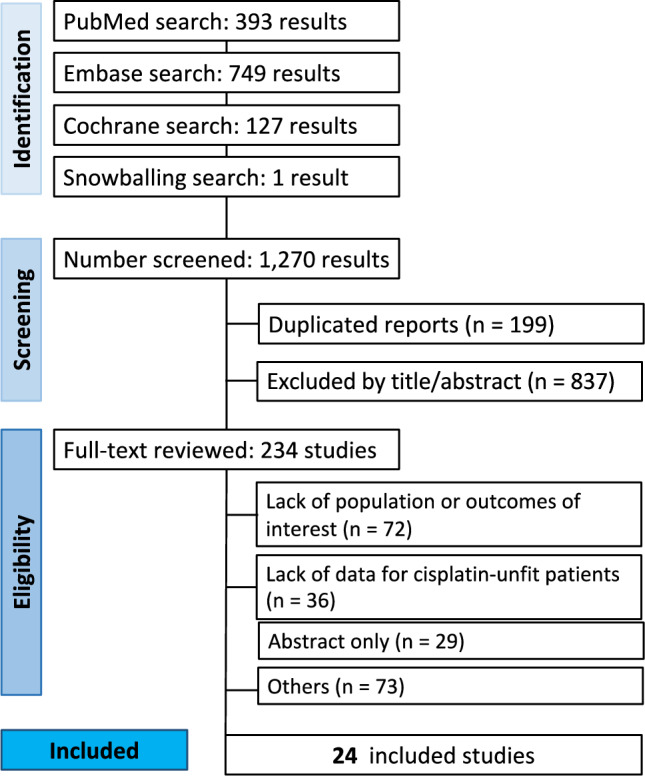
PRISMA flow diagram of study screening and selection. Blue vertical boxes indicate each stage of the screening, and the horizontal boxes present more detailed information about the process, including the steps performed in each stage

The screening process held 1270 results, of which 234 were selected for full review. Most studies lacked the population of interest or information as to cisplatin ineligibility. A list with all publications assessed can be found in Supplementary Table S7. Finally, 24 studies (18 observational studies and 6 CTs) met our eligibility criteria (Fig. 1) (Addeo et al. 2019; Agarwal et al. 2011; Beckham et al. 2020; Corry et al. 2017; Fung et al. 2020; Hamauchi et al. 2019; Han et al. 2023; Imai et al. 2022; Magnes et al. 2021; Maring et al. 2018, p. 201; Nassif et al. 2022; Patil et al. 2023; Pryor et al. 2009; Rades et al. 2023; Rambeau et al. 2017; Saigal et al. 2014; Srinivas et al. 2020; Sun et al. 2022, p. 20; Swiecicki et al. 2020; Tao et al. 2023; Ueki et al. 2023; Van Der Linden et al. 2014; Weiss et al. 2020; Ye et al. 2013).

Overall, 4450 LA HNSCC cisplatin-ineligible patients were included. Most patients were treated with cetuximab-RT (2238 patients; 50.3%), followed by carboplatin-RT (1409 patients; 31.7%). Other regimens include RT alone (312 patients; 7%); docetaxel-RT (180 patients; 4%); carboplatin-RT plus paclitaxel (118 patients; 2.7%); nimotuzumab [nimo]-RT (21 patients; 5%), pembrolizumab [pembro]-RT (96 patients; 2.1%), and carboplatin-RT combined with cetuximab (76 patients; 1.7%) (Table 1).

**Table 1:** Baseline characteristics of included studies in the systematic review

Study	Design	Location	Treatment regimen	N	Median age (range)	Male N (%)	ECOG PS scalem N (%)		Tumor location N (%)					HPV + N (%)	Smokers N (%)	Median follow-up time (range)
							0–1	≥ 2	Oral cavity	Oropharynx	Larynx	Hypopharynx	Others^a^			
Addeo et al. (2019)	Retrospective cohort	Italy	CTX-RT	64	73.7 (69–87)	46 (72)	16 (25)	48 (75)	0	31 (49)	22 (34)	11 (17)	0	5 (8)	24 (37.5)	41 (31.1–36.8)
Agarwal et al. (2011)	Retrospective cohort	India	CTX-RT	37	59 (35–87)	33 (89.2)	37 (100)	0	10 (27)	19 (51.3)	0	0	8 (21.6)	NA	27 (73)	16 (IQR: 12–23)
Beckham et al. (2020)	Retrospective cohort	USA	CB-RT (N = 74) CTX-RT (N = 61)	135	CB-RT:: 63 (44–87) CTX-RT: 66 (46–88)	CB-RT: 54 (73) CTX-RT: 36 (59)	NA	NA	0	CB-RT: 22 (29.7%) CTX-RT: 16 (26.2%)	CB-RT: 41 (55.4) CTX-RT: 35 (57.4)	CB-RT: 11 (14.9) CTX-RT: 10 (16.4)	0	0	CB-CRT: 57 (77)^b^ CTX-RT: 49 (80.3)^b^	NA
Corry et al. (2017)	Phase I/II CT	Australia	CB-RT + CTX	60	66 (42–87)	57 (95)	58 (96.7)	2 (3.3)	0	38 (63.3)	16 (26.7)	6 (10)	0	29 (83)	NA	48 (NA)
Fung et al. (2020)	Retrospective cohort	Canada	CTX-RT	115	64.5 (SD, 9.3)^c^	92 (80)	NA	NA	0	115 (100)	0	0	0	100 (87)	77 (67)	NA
Hamauchi et al. (2019)	Retrospective cohort	Japan	CB-RT (N = 29) CTX-RT (N = 18)	47	CB-RT: 74 (54–82) CTX-RT: 75 (56–83)	CB-RT: 27 (93.1) CTX-RT: 15 (83.3)	CB-RT: 26 (89.7) CTX-RT: 18 (100)	CB-RT: 3 (10.3) CTX-RT: 0	CB-RT: 2 (6.9) CTX-RT: 0	CB-RT: 11 (37.9) CTX-RT: 7 (38.9)	CB-RT: 1 (3.4) CTX-RT: 6 (33.3)	CB-RT: 15 (51.7) CTX-RT: 5 (27.8)	0	NA	CB-RT: 26 (89.7)^b^ CTX-RT: 13 (86.7)^b^	CB-RT: 60 (13.2–94.2) CTX-RT: 53.6 (25.5–62.5)
Han et al. (2023)	Retrospective cohort	USA	CB-RT + paclitaxel	65	71 (44–85)	58 (89.2)	NA	NA	0	43 (66.2)	17 (26.2)	4 (6.2)	1 (1.5)	37 (86)	49 (75.4)	29 (5–91)
Imai et al. (2022)	Retrospective cohort	Japan	CTX-RT	88	69 (48–84)	78 (89)	84 (95.5)	4 (4.5)	0	37 (42)	51 (58)^d^	d	0	10 (27)	NA	35.9 (NA)
Magnes et al. (2021)	Retrospective cohort	Austria	CTX-RT (N = 26) RT (N = 56)	82	CTX-RT: 71.5 (56–89) RT: 58.5 (43–91)	CTX-RT: 23 (88.5) RT: 45 (80.4)	CTX-RT: 17 (65.4) RT: 33 (59)	CTX-RT: 9 (34.6) RT: 5 (8.9)	NA	NA	NA	NA	NA	NA	NA	60.9 (NA)
Maring et al. (2018)	Retrospective cohort	Germany	CB-RT + paclitaxel	23	63 (43–75)	19 (82.6)	NA	NA	0	16 (70)	0	7 (30)	0	7 (30)	NA	27 (NA)
Nassif et al. (2022)	Retrospective cohort	Germany	CB-RT + paclitaxel	30	NA	22 (73.3)	NA	NA	3 (10)	9 (30)	5 (16.7)	8 (26.7)	5 (16.7)	NA	NA	41 (1–97)
Patil et al. (2023)	Phase II/III CT	India	RT (N = 176) DTX-RT (N = 180)	356	RT: 63 (26–83) DTX-RT: 61 (23–83)	RT: 153 (85) DTX-RT: 144 (81.8)	RT: 105 (59.7) DTX-RT: 91 (50.6)	RT: 71 (40.3) DTX-RT: 89 (49.4)	RT: 60 (34.1) DTX-RT: 73 (40.6)	RT: 53 (30.1) DTX-RT: 48 (26.7)	RT: 27 (15.3) DTX-RT: 24 (13.3)	RT: 30 (17) DTX-RT: 31 (17.2)	RT: 6 (2.4) DTX-RT: 4 (2.2)	RT: 2 (3.8) DTX-RT: 2 (4.2)	RT: 78 (44.3) DTX-RT: 78 (43.3)	32.4 (IQR: 26.3–42.1)
Pryor et al. (2009)	Prospective cohort	Australia	CTX-RT	13	68 (52–82)	10 (77)	NA	NA	0	5 (39)	0	5 (39)	3 (23.1)	NA	NA	NA

Rades et al. (2023)	Retrospective cohort	Germany	CB-RT	45	NA	33 (73)	NA	NA	e	26 (58)^e^	13 (29)^e^	e	6 (13)^e^	10 (29)	37 (82)	24 (0–71)
Rambeau et al. (2017)	Retrospective cohort	France	CTX-RT	88	NA	74 (84.1)	55 (62.5)	32 (36.4)	8 (9)	51 (58)	13 (14.8)	13 (14.8)	3 (3.4)	NA	83 (94.3)	9.9 (NA)
Saigal et al. (2014)	Retrospective cohort	USA	CB-RT + CTX	16	71.5 (57–90)	15 (93.7)	16 (100)	0	0	9 (56.2)	4 (25)	1 (6.25)	2 (12.5)	NA	NA	24 (1–69)
Srinivas et al. (2020)	Retrospective cohort	India	Nimo-RT	21	55 (28‐72)	17 (81)	14 (66.7)	7 (33.3)	10 (47.6)	2 (9.5)	4 (19)	3 (14.3)	2 (9.5)	NA	NA	NA
Sun et al. (2022)	Retrospective cohort	USA	CB-RT (N = 1231) CTX-RT (N = 1439)	2724	CB-RT: 64 (59–69) CTX-RT: 66 (61–72)	CB-RT: 1216 (98.8) CTX-RT: 1485 (99.5)	CB-RT: 534 (43.4) CTX-RT: 733 (49)	CB-RT: 101 (8.2) CTX-RT: 146 (9.8)	0	CB-RT: 676 (54.9) CTX-RT: 882 (59.1)	NA	NA	CB-RT: 1033 (83.9)^f^ CTX-RT: 1190 (79.7)^f^	NA	CB-RT: 549 (44.6) CTX-RT: 738 (49.3)	62 (57–66)
Swiecicki et al. (2020)	Phase II CT	USA	CTX-RT	21	65.6 (39–85)	17 (81)	17 (81)	4 (19)	2 (9.5)	16 (76,2)	0	1 (4.8%)	2 (10)	10 (48)	20 (95.2)	48 (NA)
Tao et al. (2023)	Phase II trial	Multicenter	CTX-RT (N = 66) Pembro-RT (N = 67)	133	CTX-RT: 67 (47–81) Pembro-RT: 65 (48–79)	CTX-RT: 53 (82) Pembro-RT: 59 (89)	CTX-RT: 66 (100) Pembro-RT: 67 (100)	0	CTX-RT: 5 (8) Pembro-RT: 4 (6)	CTX-RT: 40 (62) Pembro-RT: 39 (59)	CTX-RT: 9 (14) Pembro-RT: 5 (8)	CTX-RT: 11 (16.7) Pembro-RT: 18 (26.9)	0	CTX-RT: 18 (45) Pembro-RT: 18 (46)	Cet-RT: 59 (89.4) Pembro-RT: 62 (92.5)	CTX-RT: 5.8 (IQR 24.8- 26.2) Pembro-RT: 25.6 (IQR: 24.8–26.8)
Van Der Linden et al. (2014)	Retrospective cohort	NL	CTX-RT (N = 61) RT (N = 80)	141	CTX-RT: 65 (42–83) RT: 62 (40–87)	RT-CTX: 37 (60.7) RT: 61 (76.2)	CTX-RT: 27 (82)^g^ RT: 37 (95)^g^	CTX-RT: 6 (18%)^h^ RT: 2 (5)^h^	0	CTX-RT: 32 (52.5) RT: 20 (25)	CTX-RT: 6 (9.8) RT: 56 (70)	CTX-RT: 23 (37.7) RT: 4 (5)	0	NA	NA	29 (20–38)
Ueki et al. (2023)	Phase II CT	Japan	CB-RT	30	73.5 (46–85)	28 (93.3)	30 (100)	0	0	9 (30)	3 (10)	14 (47)	4 (1.3)	3 (33.3)	NA	NA
Weiss et al. (2020)	Phase II CT	Multicenter	Pembro-RT	29	63.1 (range, 39–86)^c^	28 (97)	29 (100)	0	1 (3.4)	20 (69)	3 (10.3)	2 (6.9)	3 (10.3)	14 (48.3)	18 (62)	21 (10–40)
Ye et al. (2013)	Retrospective cohort	Canada	CTX-RT	87	62 (40–89)	75 (86.2)	79 (91)	8 (9)	7 (8)	51 (59)	14 (16)	5 (6)	10 (11.5)	NA	NA	16 (NA)

Studies informing patients´ p16 + status were considered in the HPV + column; median age is given in years; median follow-up is given in months and range is presented between parenthesis unless indicated otherwise

*CB* carboplatin; *CTX* cetuximab; *CT* clinical trial; *DTX* docetaxel; ECOG *PS* Eastern Cooperative Oncology Group Perfomance Status; *HPV* + human papillomavirus positive; *IQR* interquartile range; *NA* not available; *NL* Netherlands; *N* Number of patients; *Nimo* nimotuzumab; *Pembro* Pembrolizumab; *RT* radiotherapy; *SD* standard deviation; *USA* United States

^a^other locations include unknown primary, multiple localization, and node without primitive, supraglottic larynx, oro or hypopharynx cancers, nose, paranasal sinuses, salivary glands, and lymph node metastasis

^b^data was used considering patients who smoked more than 10 packages-year

^c^values are given in mean (SD or range)

^d^this study includes patients with larynx or hypopharynx cancers

^e^refers to patients classified as non-oropharyngeal carcinoma and mixed (both oropharyngeal and non-oropharyngeal)

^f^in this study, patients' tumor site were classified as oropharynx/oral cavity, hypopharynx/larynx, or both

^g^indicates ECOG PS ≤ 1

^h^indicates ECOG PS 1–2

The majority of patients were male (4110 patients; 92.4%) and had oropharyngeal carcinoma (2343 patients; 52.7%). Tumor staging and other details regarding included population are available in Supplementary Table S6. Cisplatin-ineligibility criteria differed among studies. Common causes to consider patients unfit for cisplatin were renal and hearing impairment and poor performance status. We did not quantify the number of patients who fit each category since in many studies this information was not presented. A full description of cisplatin-ineligibility criteria is presented in Supplementary Table S8.

### Survival and response outcomes

Data for OS is presented in Table 2. In seven studies reporting median OS in patients treated with cetuximab plus RT, it ranged from 12.8 to 46 months. Median OS was superior to 43 months in two studies assessing carboplatin-RT and superior to 15 months in two studies assessing RT alone. Most studies included a limited number of patients. Nevertheless, Sun et al. 2022, the study with the greatest sample size (1493 patients on CTX-RT and 1231 on CB-RT), found significantly higher survival rates for carboplatin-based regimens compared to cetuximab. For other regimens such as nimotuzumab-RT, docetaxel-RT, and carboplatin-RT combined with paclitaxel the median OS was 21, 25.5, and 28 months, respectively. The OS at one, two, three, four, and five years according to the information available is presented in the same table.

**Table 2:** Overall survival (OS) according to the treatment regimen

(a) Median OS			
Study	Median OS (95% CI) in months	Total (N)	Treatment regimen
Addeo et al. (2019)	34 (31.1–36.8)	64	CTX-RT
Sun et al. (2022)	31.1 (12.4–87.8)	1493	CTX-RT
Rambeau et al. (2017)	12.8 (8.7–20.2)	88	CTX-RT
Magnes et al. (2021)	46.0 (9.5–82.5)	26	CTX-RT
Imai et al. (2022	25.6 (NA)	88	CTX-RT
Fung et al. (2020	34 (19–48)	115	CTX-RT
Hamauchi et al. (2019)	35.5 (NA)	18	CTX-RT
Hamauchi et al. (2019)	91.9 (NA)	29	CB-RT
Sun et al. (2022)	43.4 (15.3–123.8)	1231	CB-RT
Srinivas et al. (2020)	21 (NA)	21	Nimo-RT
Magnes et al. (2021)	58.3 (36.0–80.7)	56	RT alone
Patil et al. (2023)	15.3 (13.1–22.0)	176	RT alone
Maring et al. (2018)	28 (NA)	23	CB-RT + paclitaxel
Patil et al. (2023)	25.5 (17.6–32.5)	180	DTX-RT

(b) 1-year OS				
Study	N	Total	Proportion	Treatment regimen
Ye et al. (2013)	70	87	80.5%	CTX-RT
Swiecicki et al. (2020)	15	21	71.4%	CTX-RT
Srinivas et al. (2020)	13	21	63.7%	Nimo-RT
Weiss et al. (2020)	25	29	86.2%	Pembro-RT
Rades et al. (2023)	40	45	89%	CB-RT

(c) 2-year OS				
Study	N	Total	Proportion	Treatment regimen
Agarwal et al. (2011)	16	37	44.4%	CTX-RT
Tao et al. (2023)	36	65	55.4%	CTX-RT
Swiecicki et al. (2020)	10	21	47.6%	CTX-RT
Han et al. (2023)	58	65	88.7%	CB-RT + paclitaxel
Tao et al. (2023)	41	66	62.1%	Pembro-RT
Weiss et al. (2020)	22	29	75.9%	Pembro-RT
Patil et al. (2023)	73	176	41.7%	RT alone
Patil et al. (2023)	91	180	50.8%	DTX-RT
Rades et al. (2023)	37	45	83%	CB-RT

(d) 3-year OS				
Study	N	Total	Proportion	Treatment regimen
Magnes et al. (2021)	16	26	61.3%	CTX-RT
Rades et al. (2023)	34	45	75%	CB-RT
Nassif et al. (2022)	17	30	56.3%	CB-RT + paclitaxel
Magnes et al. (2021)	37	56	66.1%	RT alone
Saigal et al. (2014 )	11	16	71.4%	CB-RT + CTX

(e) 4-year OS				
Study	N	Total	Proportion	Treatment regimen
Corry et al. (2017)	46	60	76.7%	CTX-RT

(f) 5-year OS				
Study	N	Total	Proportion	Treatment regimen
Sun et al. (2022)	528	1231	42.9%	CB-RT
Sun et al. (2022)	507	1493	34.0%	CTX-RT
Magnes et al. (2021)	12	26	46.6%	CTX-RT
Magnes et al. (2021)	26	56	47.1%	RT alone

*OS* overall survival; *CI* confidence interval; *N* number of patients; *NA* not available; *CTX* cetuximab; *RT* radiotherapy; *CB* carboplatin; *DTX* docetaxel; *Nimo* nimotuzumab; *Pembro* pembrolizumab; *Total* indicates the total number of patients included in the study

Median PFS was reported mostly for patients on cetuximab-RT, ranging from 6.5 to 17 months across four studies (Table 3). Median PFS was 42.7 months in 29 patients on carboplatin-RT (one study), 30 months in 23 patients on carboplatin-RT combined with paclitaxel (one study), and 28.5 months in 56 patients receiving RT (one study). At 2 years, the PFS of 65 patients on carboplatin-RT combined with paclitaxel described in one study was 72.3%. For 65 patients receiving cetuximab-RT, it was 40% (one study). In two studies with 66 and 29 patients treated with pembro-RT, the PFS ranged from 42.4% to 72.4%, respectively. The PFS at 1 and 3 years is presented in Table 3.

**Table 3:** Progression-free survival (PFS) according to the treatment regimen

(a) Median PFS			
Study	Median PFS (95% CI) in months	Total (N)	Treatment regimen
Addeo et al. (2019)	14.8 (13.9–15.5)	64	CTX-RT
Hamauchi et al. (2019)	11.6 (NA)	18	CTX-RT
Rambeau et al. (2017)	6.5 (6.0–10.1)	88	CTX-RT
Magnes et al. (2021)	17 (0.0–60.3)	26	CTX-RT
Hamauchi et al. (2019)	42.7 (NA)	29	CB-RT
Maring et al. (2018)	30 (NA)	23	CB-RT + paclitaxel
Magnes et al. (2021)	28.5 (17.1–39.4)	56	RT alone

(b) 1-year PFS				
Study	N	Total	Proportion	Treatment regimen
Weiss et al. (2020)	22	29	75.9%	Pembro-RT

(c) 2-year PFS				
Study	N	Total	Proportion	Treatment regimen
Han et al. (2023)	47	65	72.3%	CB-RT + paclitaxel
Tao et al. (2023)	26	65	40%	CTX-RT
Tao et al. (2023)	28	66	42.4%	Pembro-RT
Weiss et al. (2020)	21	29	72.4%	Pembro-RT

(d) 3-year PFS				
Study	N	Total	Proportion	Treatment regimen
Nassif et al. (2022)	21	30	68.9%	CB-RT + paclitaxel
Saigal et al. (2014)	6	16	39.7%	CB-RT + CTX

*PFS* progression-free survival; *CI* confidence interval; *N* number of patients; *NA* not available; *CTX* cetuximab; *RT* radiotherapy; *CB* carboplatin; *Pembro* pembrolizumab; *Total* indicates the total number of patients included in the study

The 1-year DFS was reported in two studies (21 and 87 patients in each) assessing cetuximab-RT and was superior to 45% in both studies (Supplementary Table S9). At two years, two studies with 21 and 37 patients reported the DFS, varying between 29.5% and 38.1%. For 176 patients treated with RT alone, it was 30.3% (one study). In one study, the 2-year DFS of 180 patients on docetaxel-RT was 42.2% (Supplementary Table S9).

The LRC at one year or 15 months was described in three studies including the following regimens: cetuximab-RT (143 patients), pembro-RT (57 patients), and carboplatin-RT (45 patients) (Supplementary Table S10). A rate of 78% was seen for patients on carboplatin-RT. Patients on pembro-RT had a 59.6% LRC. The group on CTX-RT presented with rates varying between 58.9% and 71.3%. At two years, one study with 45 patients on carboplatin-RT reported a higher rate of 69% compared to 35.5% for 37 patients treated with cetuximab-RT (Supplementary Table S10). The 3-year LRC was higher for 30 patients on carboplatin-RT combined with paclitaxel compared with other regimens, as shown in Supplementary Table S10.

The ORR, DCR, and the incidence of distant recurrence and locoregional failure according to the regimen used are presented in Tables S11 and S12, respectively.

### Adverse events

For two studies on carboplatin-based regimens, the most common AEs were dry mouth, RT dermatitis, and mucositis. The majority of them were grade 1–2, except for mucositis, in which both studies reported high rates of grade 3 events (Supplementary Table S13A).

Eight studies reported skin toxicity in patients treated with cetuximab-RT, most of them grade 1–2. Four studies described important rates of grade 3 or higher RT dermatitis. In some of them, grade 3 AEs accounted for more than 50% of all events. Dysphagia was described in four studies, with most events being grade 1–2. Among eight studies exploring CTX-associated mucositis, three reported a high incidence (above 50%) of grade 3 AEs (Supplementary Table S13B).

One study explored AEs of 179 patients on docetaxel-RT. Skin toxicity, mucositis, and dysphagia were often reported. For skin toxicity, most events were grade 1–2. However, important rates of grade 3 or higher mucositis and dysphagia were observed (Supplementary Table S13C).

Pembro-RT and nimo-RT were assessed in two and one studies, respectively (Supplementary Table S13D e S13E). Anemia, mucositis, dysphagia, and diarrhea were frequently observed in patients receiving either regimen. For the group receiving pembro-RT, most AEs were grade 1–2. No grade 3 or higher AEs were observed in patients on nimo-RT. For other regimens, the toxicity profile is shown in Supplementary Table S13.

### Quality assessment

Overall, six out of 22 non-randomized studies were judged to be at high risk of bias (Supplementary Table S14) (Addeo et al. 2019; Hamauchi et al. 2019; Rambeau et al. 2017; Saigal et al. 2014; Srinivas et al. 2020; Van Der Linden et al. 2014). Most of them lacked adjustments for confounding factors, failing to meet the criteria for the first domain. All other observational studies were adjusted for cofounders and judged at moderate risk of bias, as well as the phase II CTs (Agarwal et al. 2011; Beckham et al. 2020; Corry et al. 2017; Fung et al. 2020; Han et al. 2023; Imai et al. 2022; Magnes et al. 2021; Maring et al. 2018, p. 201; Nassif et al. 2022; Pryor et al. 2009; Rades et al. 2023; Sun et al. 2022; Swiecicki et al. 2020; Ueki et al. 2023; Weiss et al. 2020; Ye et al. 2013). The two randomized clinical trials met most criteria for all domains and were determined to be at low risk of bias (Supplementary Table S14) (Patil et al. 2023; Tao et al. 2023).

## Discussion

To our knowledge, this is the first systematic review evaluating treatment alternatives for cisplatin-ineligible LA HNSCC patients. Median OS ranged from 12.8 to 46 months in seven studies including patients treated with cetuximab-RT. The median OS was superior to 40 months in two studies assessing carboplatin-RT and superior to 15 months in two studies assessing RT alone. For other regimens such as nimotuzumab-RT, docetaxel-RT, and carboplatin-RT combined with paclitaxel, the median OS was superior to 21 months. Median PFS was reported in four studies on patients on cetuximab-RT and it ranged from 6.5 to 17 months. Median PFS was superior to 20 months in patients treated with carboplatin-RT, carboplatin-RT combined with paclitaxel, or RT alone.

Cisplatin-ineligibility can be classified into two groups: absolute and relative contraindications (Szturz et al. 2019). Some of the absolute contraindications include impaired renal and hearing function and peripheral neuropathy (Kim et al. 2023; Szturz et al. 2019). Relative contraindications comprise patients who may not derive benefit from cisplatin due to individual factors (*e.g.* age, performance status, weight loss, previous cardiovascular disease) (Kim et al. 2023; Szturz et al. 2019). Reflecting the universal lack of consensus regarding cisplatin-ineligibility criteria, the studies included in this systematic review were heterogeneous in classifying patients as non-eligible for cisplatin.

International guidelines are vague in recommending treatment options for the LA HNSCC cisplatin-unfit population due to little evidence to support alternative regimens (Koyfman et al. 2019; Machiels et al. 2020). Patients unsuitable for cisplatin are frequently treated based on extrapolated data from studies assessing non-cisplatin regimens in populations eligible for cisplatin and who do not express the large comorbidity burden of unfit patients (Haddad et al. 2023). Accordingly, cetuximab raised as an alternative regimen over the standard of care (SOC) cisplatin-based CRT since its approval in the US in 2011 (Porceddu et al. 2020). The addition of CTX to RT was investigated in a phase III trial conducted by Bonner et al*.*, showing a greater benefit for patients in the combination group compared to RT alone (Bonner et al. 2006). However, some authors support RT alone, especially intensity-modulated radiotherapy, as an alternative for such a population (Haddad et al. 2023). Multiple trials have shown the inferior efficacy of cetuximab-RT compared to SOC (Machiels et al. 2020; NCCN Clinical Practice Guidelines in Oncology. Head and Neck Cancer., n.d.). Yet, with a more acceptable AE profile compared to cisplatin, the combination of cetuximab and RT remains a reasonable option for LA HNSCC patients with contraindications to cisplatin (Addeo et al. 2019; Rambeau et al. 2017).

Carboplatin plus RT is another alternative for non-eligible patients. Although there is no phase III trial assessing CTX-RT *versus* CB-RT in cisplatin-unfit patients, retrospective studies support carboplatin over cetuximab (Sun et al. 2022). In a large US veteran cohort study conducted in 1231 patients treated with carboplatin and 1493 with cetuximab regimens, carboplatin was associated with a 15% OS benefit compared to cetuximab (Sun et al. 2022). In this study, most patients on carboplatin received a combination of carboplatin-RT and docetaxel, a derivative of paclitaxel. Accordingly, the phase III study conducted by Patil et al*.* on 180 patients treated with docetaxel plus RT compared to 176 patients on RT alone found an improvement in OS, DFS, and LRC in favor of the docetaxel-arm (Patil et al. 2023). Furthermore, the use of docetaxel plus RT and cetuximab as adjuvant treatment is recommended with a category 2B by NCCN guidelines for the LA HNSCC cisplatin-ineligible population (NCCN Clinical Practice Guidelines in Oncology. Head and Neck Cancer., n.d.).

Immunotherapy agents are currently emerging for the treatment of HNC in various stages (Tao et al. 2023; Weiss et al. 2020). Previous studies support the benefit of anti-PD1 agents in the recurrent or metastatic setting (Burtness et al. 2019). In the cisplatin-ineligibility scenario, a phase III trial recently explored the role of ICI combined with RT for LA HNSCC (Tao et al. 2023). The study conducted by Tao et al*.* compared 66 patients on Pembro-RT with 65 patients on cetuximab-RT (Tao et al. 2023). The OS, PFS, and LRC were similar between groups (Tao et al. 2023). Nevertheless, the AEs rates were significantly lower in the pembro-arm, suggesting that this combination may be as efficient as cetuximab-based regimens with a milder toxicity profile. Data from ongoing studies analyzing other combinations are awaited, such as the phase III REACH studying avelumab plus CTX plus RT *versus* SOC (NCT02999087) and NANORAY-312, a phase III trial assessing NBTXR3 in combination with RT and CTX for cisplatin-unfit patients (NCT04892173).

It is noteworthy to mention that all these regimens may have different responses and tolerability profiles according to tumor location, risk stratification, and individual factors (Haddad et al. 2023; Porceddu et al. 2020). For instance, human papillomavirus (HPV)-negative patients are known to present with poor outcomes, both on cisplatin-based or alternative regimens (Haddad et al. 2023; Kim et al. 2023, p. 2; Porceddu et al. 2020). These patients are often older and present with an even higher burden of comorbidities. Therefore, different factors such as tumor location and risk, sociodemographic characteristics, and adverse-events profile should be carefully considered in treatment decisions for HNSCC cisplatin non-eligible patients.

This study has limitations. First, network meta-analysis is the preferred method to compare multiple interventions in a specific population (Faltinsen et al. 2018). The limited high-quality evidence and important heterogeneity among studies prevented us from applying meta-analyses. The study’s design, follow-up time, cisplatin ineligibility criteria, outcomes and ‘locally advanced head and neck cancer’ definition, and treatment schedules also differed in included studies. For some treatment regimens, a limited number of studies were available. Due to the unavailability of data from most studies, data stratified on tumor location and risk stratification could not be presented.

This large systematic review includes over 4000 cisplatin-unfit LA HNSCC patients and covers a range of alternative therapies for this population, including cetuximab-RT, carboplatin-RT, carboplatin-RT combined with cetuximab or paclitaxel, RT alone, docetaxel-RT, and pembro or nimo-RT. The response, survival, and safety data presented offer essential insights and guidance for clinicians treating this group. Yet, the lack of consensus on cisplatin ineligibility criteria adds important complexity to the management of these patients. Thus, future studies should focus on establishing cisplatin ineligibility criteria and exploring tolerable but effective alternative strategies for this population. Importantly, patients should be stratified according to relevant clinicopathological factors (*i.e.* tumor location, HPV status, patient’s age, and performance status).

## Conclusions

To the best of our knowledge, this is the first systematic review evaluating treatment alternatives for cisplatin-ineligible LA HNSCC patients. This study gathers extensive information regarding treatment strategies available for cisplatin-non-eligible LA HNSCC patients within CTs and in the real-world setting. Our findings support the use of several available options such as cetuximab-RT, carboplatin-RT, carboplatin-RT combined with cetuximab or paclitaxel, RT alone, docetaxel-RT, and pembro or nimo-RT for cisplatin-unfit LA HNSCC patients. Nonetheless, we highlight the limited evidence to guide treatment choice and the urgent need for clinical studies assessing new treatment approaches in this population.

## Supplementary Information

Below is the link to the electronic supplementary material.

Supplementary file1 (PDF 871 KB)

## Data Availability

All research data presented in this study is accessible upon request to the corresponding author.
