# Supplementary material for: Treatment options for cisplatin-ineligible patients with locally advanced head and neck squamous cell carcinoma: a systematic review
Source: J Cancer Res Clin Oncol. 2024 Aug 2;150(8):379. doi: 10.1007/s00432-024-05887-z (PMC11297068; doi:10.1007/s00432-024-05887-z)
Supplement: Supplementary file 1 — Supplementary file1 (PDF 871 KB) [file 432_2024_5887_MOESM1_ESM.pdf]

# Treatment Options for Cisplatin-Ineligible Patients with Locally Advanced Head and Neck Squamous Cell Carcinoma: A Systematic Review

***Supplementary Material***

Isabella Michelon[1], Gilca Costa Nachtigal[2] MD, MSc, Maria Inez Dacoregio[3], Ana Cristina Beitia Kraemer Moraes[4], MD, Mauricio Moraes[5], MD, Lívia Silva Piva[5], Catiara Terra da Costa[6], PhD, Rafael Guerra Lund[6], PhD, Douver Michelon[6], PhD

[1] Department of Medicine, Catholic University of Pelotas, Pelotas, Brazil.
isabella.michelon20@gmail.com

[2] Department of Internal Medicine, Federal University of Pelotas Teaching Hospital (EBSERH), Pelotas, Brazil. gilcacn@gmail.com

[3] Department of Medicine, University of Centro Oeste, Guarapuava, Brazil.
m.dacoregio2707@gmail.com

[4] Department of Surgery, Faculty of Medicine, Catholic University of Pelotas, Pelotas, Brazil.
Pelotas Dental School, Graduate Program in Dentistry, Federal University of Pelotas, Pelotas, Brazil.
anacristinabkmoraes@gmail.com

[5] Department of Medicine, Federal University of Pelotas, Pelotas, Brazil. mumana74@gmail.com, liviapivamed@gmail.com

[6] Graduate Program in Dentistry, School of Dentistry, Federal University of Pelotas, Pelotas, RS, Brazil. catiaraorto@gmail.com, rglund@ufpel.edu.br, douvermichelon@gmail.com

**Corresponding author:**
Douver Michelon, PhD
Graduate Program in Dentistry, School of Dentistry, Federal University of Pelotas, 96015560, Pelotas, Brazil. douvermichelon@gmail.com

**Table of contents**

*Table S1: Preferred Reporting Items for Systematic Reviews and Meta-Analysis (PRISMA) Checklist for the Manuscript (A), and for the Abstract (B).* 4

(A) PRISMA checklist for the manuscript. 4

(B) PRISMA checklist for the abstract. 5

*Table S2: Full search strategy used in each database: (A) PubMed; (B) Embase; and (C) Cochrane.* 6

(A) PubMed Search 6

(B) Embase Search 6

(C) Cochrane Search 6

*Table S3: Eligibility criteria and treatment schedule details of studies included in this systematic review.* 7

*Table S4: Definition of outcomes according to the information available in studies included in this systematic review.* 12

*Table S5: Other outcomes not included in main tables.* 14

*Table S6: Detailed information regarding tumor staging based on available information of studies included in this systematic review.* 18

*Table S7: List of excluded studies after a comprehensive review.* 20

*Table S8: Cisplatin ineligibility criteria according to the information available in studies included in the systematic review.* 29

*Table S9: Disease-free survival (DFS) at 1 year (A); and 2 years (B), according to the treatment regimen.* 32

(A) 1-year DFS 32

(B) 2-year DFS 32

*Table S10: Locoregional control rate (LRC) at 1 year/15 months (A); 2 years (B), 3 years (C); and 4 years (D), according to the treatment regimen.* 33

(A) LRC at one year or 15-months 33

(B) 2-year LRC 33

(C) 3-year LRC 33

(D) 4-year LRC 33

*Table S11: Incidence of distant recurrence/metastases (A); and locoregional failure (B), according to the regimen used.* 34

(A) Incidence of distant recurrence or metastases according to the regimen used. 34

(B) Locoregional failure according to the regimen used. 34

*Table S12: Objective response rate (A); and disease control rate (B) according to the regimen used.* 35

(A) Objective response rate (ORR) according to the regimen used. 35

(B) Disease control rate (DCR) according to the regimen used. 35

*Table S13: Incidence of commonly reported adverse events among patients treated with carboplatin-RT (A); cetuximab-RT (B); docetaxel-RT (C); pembrolizumab-RT (D); nimotuzumab-RT (E); carboplatin-RT plus paclitaxel (F); and carboplatin-RT plus cetuximab (G).* 36

**(A) Carboplatin-RT** 36

**(B) Cetuximab-RT** 37

**(C) Docetaxel-RT** 39

**(D) Pembrolizumab-RT** 40

**(E) Nimotuzumab-RT** 41

**(F) Carboplatin-RT plus paclitaxel** 42

**(G) Carboplatin-RT plus cetuximab** 43

*Table S14: Quality assessment for studies included in this systematic review using: risk of bias summary for non-randomized studies (ROBINS-I) tool for retrospective cohorts and prospective studies (A), and risk-of-bias 2 tool for randomized clinical trials (RoB 2) (B).* 44

**(A) Risk of bias summary for non-randomized studies (ROBINS-I).** 44

**(B) Risk of bias summary for randomized studies (RoB 2).** 46

**TABLES**

**Table S1: Preferred Reporting Items for Systematic Reviews and Meta-Analysis (PRISMA) Checklist for the Manuscript (A), and for the Abstract (B).**

**(A) PRISMA checklist for the manuscript.**

| Section and Topic | Item # | Checklist item | Location where item is reported |
|---|---|---|---|
| **TITLE** | | | |
| Title | 1 | Identify the report as a systematic review. | Page 1 |
| **ABSTRACT** | | | |
| Abstract | 2 | See the PRISMA 2020 for Abstracts checklist. | Table S1 (B) |
| **INTRODUCTION** | | | |
| Rationale | 3 | Describe the rationale for the review in the context of existing knowledge. | Pages 3 |
| Objectives | 4 | Provide an explicit statement of the objective(s) or question(s) the review addresses. | Pages 3 |
| **METHODS** | | | |
| Eligibility criteria | 5 | Specify the inclusion and exclusion criteria for the review and how studies were grouped for the syntheses. | Page 4 |
| Information sources | 6 | Specify all databases, registers, websites, organisations, reference lists and other sources searched or consulted to identify studies. Specify the date when each source was last searched or consulted. | Page 4 |
| Search strategy | 7 | Present the full search strategies for all databases, registers and websites, including any filters and limits used. | Table S2 |
| Selection process | 8 | Specify the methods used to decide whether a study met the inclusion criteria of the review, including how many reviewers screened each record and each report retrieved, whether they worked independently, and if applicable, details of automation tools used in the process. | Pages 4, 5 |
| Data collection process | 9 | Specify the methods used to collect data from reports, including how many reviewers collected data from each report, whether they worked independently, any processes for obtaining or confirming data from study investigators, and if applicable, details of automation tools used in the process. | Page 4, 5 |
| Data items | 10a | List and define all outcomes for which data were sought. Specify whether all results that were compatible with each outcome domain in each study were sought (e.g. for all measures, time points, analyses), and if not, the methods used to decide which results to collect. | Page 5 |
| | 10b | List and define all other variables for which data were sought (e.g. participant and intervention characteristics, funding sources). Describe any assumptions made about any missing or unclear information. | Page 5 |
| Study risk of bias assessment | 11 | Specify the methods used to assess risk of bias in the included studies, including details of the tool(s) used, how many reviewers assessed each study and whether they worked independently, and if applicable, details of automation tools used in the process. | Page 5 |
| Effect measures | 12 | Specify for each outcome the effect measure(s) (e.g. risk ratio, mean difference) used in the synthesis or presentation of results. | NA |
| Synthesis methods | 13a | Describe the processes used to decide which studies were eligible for each synthesis (e.g. tabulating the study intervention characteristics and comparing against the planned groups for each synthesis (item #5)). | NA |
| | 13b | Describe any methods required to prepare the data for presentation or synthesis, such as handling of missing summary statistics, or data conversions. | NA |
| | 13c | Describe any methods used to tabulate or visually display results of individual studies and syntheses. | Page 5 |
| | 13d | Describe any methods used to synthesize results and provide a rationale for the choice(s). If meta-analysis was performed, describe the model(s), method(s) to identify the presence and extent of statistical heterogeneity, and software package(s) used. | NA |
| | 13e | Describe any methods used to explore possible causes of heterogeneity among study results (e.g. subgroup analysis, meta-regression). | NA |
| | 13f | Describe any sensitivity analyses conducted to assess robustness of the synthesized results. | NA |
| Reporting bias assessment | 14 | Describe any methods used to assess risk of bias due to missing results in a synthesis (arising from reporting biases). | NA |
| Certainty assessment | 15 | Describe any methods used to assess certainty (or confidence) in the body of evidence for an outcome. | NA |
| **RESULTS** | | | |
| Study selection | 16a | Describe the results of the search and selection process, from the number of records identified in the search to the number of studies included in the review, ideally using a flow diagram. | Pages 5 |
| | 16b | Cite studies that might appear to meet the inclusion criteria, but which were excluded, and explain why they were excluded. | Table S7 |
| Study characteristics | 17 | Cite each included study and present its characteristics. | Table 1 |
| Risk of bias in studies | 18 | Present assessments of risk of bias for each included study. | Table S11 |
| Results of individual studies | 19 | For all outcomes, present, for each study: (a) summary statistics for each group (where appropriate) and (b) an effect estimate and its precision (e.g. confidence/credible interval), ideally using structured tables or plots. | NA |
| Results of syntheses | 20a | For each synthesis, briefly summarise the characteristics and risk of bias among contributing studies. | Page 10 |

| Section and Topic | Item # | Checklist item | Location where item is reported |
|---|---|---|---|
| | 20b | Present results of all statistical syntheses conducted. If meta-analysis was done, present for each the summary estimate and its precision (e.g. confidence/credible interval) and measures of statistical heterogeneity. If comparing groups, describe the direction of the effect. | NA |
| | 20c | Present results of all investigations of possible causes of heterogeneity among study results. | NA |
| | 20d | Present results of all sensitivity analyses conducted to assess the robustness of the synthesized results. | NA |
| Reporting biases | 21 | Present assessments of risk of bias due to missing results (arising from reporting biases) for each synthesis assessed. | NA |
| Certainty of evidence | 22 | Present assessments of certainty (or confidence) in the body of evidence for each outcome assessed. | NA |
| **DISCUSSION** | | | |
| Discussion | 23a | Provide a general interpretation of the results in the context of other evidence. | Page 8 |
| | 23b | Discuss any limitations of the evidence included in the review. | Page 10 |
| | 23c | Discuss any limitations of the review processes used. | Page 10 |
| | 23d | Discuss implications of the results for practice, policy, and future research. | Page 10 |
| **OTHER INFORMATION** | | | |
| Registration and protocol | 24a | Provide registration information for the review, including register name and registration number, or state that the review was not registered. | Page 4 |
| | 24b | Indicate where the review protocol can be accessed, or state that a protocol was not prepared. | Page 4 |
| | 24c | Describe and explain any amendments to information provided at registration or in the protocol. | Page 10 |
| Support | 25 | Describe sources of financial or non-financial support for the review, and the role of the funders or sponsors in the review. | Page 10 |
| Competing interests | 26 | Declare any competing interests of review authors. | Page 10 |
| Availability of data, code and other materials | 27 | Report which of the following are publicly available and where they can be found: template data collection forms; data extracted from included studies; data used for all analyses; analytic code; any other materials used in the review. | Page 10 |

**(B) PRISMA checklist for the abstract.**

| Section and Topic | Item # | Checklist item | Reported (Yes/No) |
|---|---|---|---|
| **TITLE** | | | |
| Title | 1 | Identify the report as a systematic review. | Yes |
| **BACKGROUND** | | | |
| Objectives | 2 | Provide an explicit statement of the main objective(s) or question(s) the review addresses. | Yes |
| **METHODS** | | | |
| Eligibility criteria | 3 | Specify the inclusion and exclusion criteria for the review. | Yes |
| Information sources | 4 | Specify the information sources (e.g. databases, registers) used to identify studies and the date when each was last searched. | Yes |
| Risk of bias | 5 | Specify the methods used to assess risk of bias in the included studies. | No |
| Synthesis of results | 6 | Specify the methods used to present and synthesise results. | Yes |
| **RESULTS** | | | |
| Included studies | 7 | Give the total number of included studies and participants and summarise relevant characteristics of studies. | Yes |
| Synthesis of results | 8 | Present results for main outcomes, preferably indicating the number of included studies and participants for each. If meta-analysis was done, report the summary estimate and confidence/credible interval. If comparing groups, indicate the direction of the effect (i.e. which group is favoured). | Yes |
| **DISCUSSION** | | | |
| Limitations of evidence | 9 | Provide a brief summary of the limitations of the evidence included in the review (e.g. study risk of bias, inconsistency and imprecision). | No |
| Interpretation | 10 | Provide a general interpretation of the results and important implications. | Yes |
| **OTHER** | | | |
| Funding | 11 | Specify the primary source of funding for the review. | Yes |
| Registration | 12 | Provide the register name and registration number. | No |

NA: not available

**Table S2: Full search strategy used in each database: (A) PubMed; (B) Embase; and (C) Cochrane.**

| | |
|---|---|
| **(A) PubMed Search** | (HNSCC OR “head and neck” OR “head and neck squamous cell carcinoma“ OR “head and neck cancer” OR “squamous cell carcinoma of the head and neck”) AND ((cisplatin OR chemotherapy OR chemotherapies OR platinum OR CDDP OR “cis platinum” OR “cis-diamminedichloroplatinum(II)” OR “platinum diamminodichloride” OR “cis-platinum” OR “cis platinum” OR dichlorodiammineplatinum OR “cis-diamminedichloroplatinum” OR “cis diamminedichloroplatinum” OR “cis-dichlorodiammineplatinum(II)” OR platino OR platinol OR biocisplatinum OR platidiam) AND (unfit OR ineligible OR “not eligible” OR contraindication OR ineligibility OR unsuitable)) |
| **(B) Embase Search** | ('hnscc'/exp OR hnscc OR 'head and neck' OR 'head and neck squamous cell carcinoma'/exp OR 'head and neck squamous cell carcinoma' OR 'head and neck cancer'/exp OR 'head and neck cancer' OR 'squamous cell carcinoma of the head and neck'/exp OR 'squamous cell carcinoma of the head and neck') AND ('cisplatin'/exp OR cisplatin OR 'chemotherapy'/exp OR chemotherapy OR chemotherapies OR 'platinum'/exp OR platinum OR 'cddp'/exp OR cddp OR 'cis-diamminedichloroplatinum(ii)'/exp OR 'cis-diamminedichloroplatinum(ii)' OR 'platinum diamminodichloride' OR 'cis-platinum'/exp OR 'cis-platinum' OR 'cis platinum'/exp OR 'cis platinum' OR 'dichlorodiammineplatinum'/exp OR dichlorodiammineplatinum OR 'cis-diamminedichloroplatinum'/exp OR 'cis-diamminedichloroplatinum' OR 'cis diamminedichloroplatinum'/exp OR 'cis diamminedichloroplatinum' OR 'cis-dichlorodiammineplatinum(ii)'/exp OR 'cis-dichlorodiammineplatinum(ii)' OR platino OR 'platinol'/exp OR platinol OR 'biocisplatinum'/exp OR biocisplatinum OR 'platidiam'/exp OR platidiam) AND (unfit OR ineligible OR 'not eligible' OR 'contraindication'/exp OR contraindication OR ineligibility OR unsuitable) |
| **(C) Cochrane Search** | (HNSCC OR "head and neck" OR "head and neck squamous cell carcinoma" OR "head and neck cancer" OR "squamous cell carcinoma of the head and neck") AND ((cisplatin OR platinum OR chemotherapy OR chemotherapies) AND (unfit OR ineligible OR “not eligible” OR contraindication OR ineligibility OR unsuitable)) |

**Table S3: Eligibility criteria and treatment schedule details of studies included in this systematic review.**

| Study | Design | Treatment regimen | Main inclusion criteria | Main exclusion criteria | Treatment schedule and details |
|---|---|---|---|---|---|
| **Addeo 2019** | Retrospective cohort | CTX-RT | age 65 years or older; an Eastern Cooperative Oncology Group (ECOG) performance status of 0–3; oropharynx, larynx, or hypopharynx unresectable or inoperable tumor; and informed consent. | history of malignancy in the last 2 years, previous RT or chemotherapy for HNC, nonmelanomatous skin cancer, or localized early-stage prostate cancer, with patients continually disease-free, or cancers that are not felt to influence treatment or the life expectancy of the patient, clinical evidence of active infection, overt systemic metastatic disease, use of experimental medications within the last 4 weeks prior to day 1, and allergic reaction to cetuximab. | **CTX:** weekly cetuximab initially at a dose of 400 mg/m2 of body surface area and then at 250 mg weekly during the whole RT.<br>**RT:** high-dose radiotherapy; one 2-Gy fraction per day, 5 days a week, for a total of 70 Gy. |
| **Agarwal 2011** | Retrospective cohort | CTX-RT | patients with LASCCHN planned for curative intent treatment but unsuitable or ineligible for platinum-based concurrent chemo-radiotherapy based on decision-making in a multi-disciplinary head-neck cancer joint clinic were considered eligible for inclusion. | patients treated with palliative intent or for locoregionally recurrent or metastatic disease were excluded from the study cohort. | **CTX::** weekly cetuximab initially at a dose of 400 mg/m2 of body surface area and then at 250 mg weekly during the whole RT.<br>**RT:** 3D or conventionally fractionated (2 Gy/fraction, 5 fractions/week). |
| **Beckham 2019** | Retrospective cohort | CB-RT (N=74)<br>CTX-RT (N=61) | three hundred and sixteen consecutive patients with treatment- naïve stage III-IVB HNSCC (AJCC 7th edition) were identified from Memorial Sloan Kettering Cancer Center between 2002 and 2013 and The Ohio State University James Cancer Center between 2009 and 2015 who received definitive RT with concurrent cisplatin, carboplatin-based regimens or cetuximab. | patients who received induction chemotherapy were not included. | **RT:** range between 67.84 and 72.0 Gy |
| **Corry 2017** | Phase I/II trial | CB-RT+CTX | patients with locally advanced (stage III or IV), biopsy-proven HNSCC of the oropharynx, larynx, or hypopharynx who were fit for curable treatment but unfit for cisplatin chemotherapy. | evidence of distant metastatic disease confirmed by a computed tomography (CT) of the chest and abdomen fluorodeoxyglucose (FDG)-positron emission tomography (PET)/CT. | **CTX:** weekly cetuximab initially at a dose of 400 mg/m2 of body surface area and then at 250 mg weekly during the whole RT.<br>**CB:** weekly at an AUC of 2mg/ml/min.<br>**RT:** IFB regimen (66 Gy in 35 fractions over 5 weeks). |
| **Fung 2020** | Retrospective cohort | CTX-RT | patients with stage III, IVA/B oropharyngeal carcinoma treated with either cisplatin or cetuximab given concurrently with radiation at two tertiary cancer centers within the province of Alberta, Canada between 2006 and 2016. | patients whose treatment included surgery (n = 212), had radiation alone (n = 32), or received systemic therapies other than cisplatin or cetuximab (n = 75) were excluded. Six patients received both cisplatin and cetuximab and two patients had unknown systemic therapy and were also excluded. | **CTX:** weekly cetuximab initially at a dose of 400 mg/m2 of body surface area and then at 250 mg weekly during the whole RT.<br>**RT:** Patients receiving definitive RT were treated with 70 Gy in 33 to 35 fractions. |

| | | | | | |
|---|---|---|---|---|---|
| **Hamauchi 2019** | Retrospective cohort | CB-RT (N=29)<br>CTX-RT (N=18) | (1) pathologically proven squamous cell carcinoma of the oral cavity, oropharynx, hypopharynx, or larynx; (2) ineligibility for cisplatin-based treatment because of the presence of ≥1 of the factors of age>76 years, renal impairment [creatinine clearance (CCr)<60 mL/min], cardiac dysfunction (history of unstable angina pectoris, myocardial infarction, or chronic heart failure), neurologic impairment (peripheral neuropathy or hearing impairment), or PS 2. | no distant metastatic disease; no active concomitant malignancy; and no prior RT or surgery. The present study also included the additional criterion of no prior induction chemotherapy (ICT). | **CB:** administered tri-weekly (AUC, 4–6 on days 1, 22, and 43) or once weekly (AUC, 1.5–2 on days 1, 8, 15, 22, 29, 36, and 43) at their physician's discretion.<br>**CTX:** weekly cetuximab initially at a dose of 400 mg/m2 of body surface area and then at 250 mg weekly during the whole RT.<br>**RT:** 3D conformal RT; planned total radiation dose was 70 Gy (2 Gy per day, 5 days per week). Three-dimensional conformal RT was delivered through a linear accelerator with a 6-MV X-ray. |
| **Han 2023** | Retrospective cohort | CB-RT+paclitaxel | patients with HNSCC treated at Memorial Sloan Kettering Cancer Center (MSKCC) from 2013 to 2021 that received definitive RT with weekly carboplatin and paclitaxel for oropharynx, larynx, hypopharynx, and unknown primary cancers. | we excluded patients who received induction chemotherapy or re-irradiation and patients who had multiple synchronous primary malignancies. Patients with prior invasive malignancies other than non-melanomatous skin cancer were excluded unless they remained disease free for 3 years or if the 5-year cure rate from treatment was greater than 90%. | **CB-PTX:** median doses of carboplatin and paclitaxel were AUC 1 and 40 mg/m2, respectively.<br>**RT:** median radiation dose received was 70 Gy. |
| **Imai 2022** | Retrospective cohort | CTX-RT | patients eligible for inclusion in this single-center retrospective study were those with stage III-IVB LA-SCCHN with primary sites in the hypopharynx, larynx, and oropharynx who received radiotherapy plus cetuximab at primary treatment between July 2013 and October 2018. | patients with cancer in the nasal cavity, oral cancer, and salivary gland cancer were excluded. Stage I-II and metastatic or recurrent cases were also excluded. Patients were also ineligible if they had undergone surgery or had previously received radiotherapy for head and neck cancer. | **CTX:** weekly cetuximab initially at a dose of 400 mg/m2 of body surface area and then at 250 mg weekly during the whole RT.<br>**RT:** the planned total radiation dose was 70 Gy (2 Gy/day, 5 days/week), delivered using IMRT or 3D conformal radiotherapy |
| **Magnes 2021** | Retrospective cohort | CT-RT (N=26)<br>RT (N=56) | patients who were treated with postoperative radiotherapy alone or in combination with cisplatin or cetuximab. | NA | **NA** |
| **Maring 2018** | Retrospective cohort | CB-RT+paclitaxel | Inclusion criteria for our study were: no pre-treatment of LASCCHN in oropharynx and hypopharynx, UICC stage III or IV without distant metastasis, cisplatin or carboplatin/paclitaxel (CarboTaxol) CTX regimens, completion of treatment course and a minimum follow-up time of 3 months. | NA | **CB-PTX:** carboplatin (AUC1) 40 mg/m2 body surface area and paclitaxel.<br>**RT:** conventionally fractionated IMRT. The median total dose and median fraction dose for the RT were 66.6 Gy (range 59.4–72) and 1.8 Gy, respectively. |
| **Nassif 2022** | Retrospective cohort | CB-RT+paclitaxel | data were collected from patients who underwent simultaneous RCT as part of the multimodal treatment of advanced head and neck squamous cell carcinoma at our institution between May 2012 and December 2017. Patient-related demographical and treatment data were gathered from records and analysed for each group separately as well as for all patients as a whole group. | NA | **CB-PTX-RT:** patients were treated with AUC2+paclitaxel (45 mg/m2 BSA) |
| **Patil 2023** | Phase II/III trial | RT (N=176)<br>DTX-RT (N=180) | adult patients (age > 18 years) with nonmetastatic, LAHNSCC arising from the oral cavity, oropharynx, hypopharynx, larynx, or carcinoma of unknown primary with neck nodes warranting either definitive or adjuvant chemoradiation, with an Eastern Cooperative Oncology Group performance status (ECOG PS) of 0-2, who were cisplatin-ineligible were selected for this study. | NA | **DTX:** administered in a dose of 15 mg per m2 once weekly. The first dose was administered within 5 days of the start of radiation.<br>**RT:** patients treated in definitive settings received a dose of 70 Gy, 2 Gy/fraction, 5 days a week, over 7 weeks. Those in adjuvant settings received 60 Gy, 2 Gy/fraction, 5 days a week over 6 weeks. It was delivered using a standard conventional two-dimensional or 3D conformal or IMRT |

| | | | | | |
|---|---|---|---|---|---|
| **Pryor 2009** | Prospective cohort | CTX-RT | data were prospectively collected on the first 13 consecutive patients with LAHNSCC (stage III, IVa,b) undergoing curative concurrent cetuximab and RT at our institution between August 2007 and May 2008. Patients were deemed fit for radical RT with an Eastern Cooperative Oncology Group (ECOG) performance sta- tus of 0–1, but deemed unsuitable for concurrent cisplatin-based chemotherapy by the medical oncologist due to pre-existing renal or hearing impairment or due to a combination of other medical comorbidities. | NA | **CTX:** weekly cetuximab initially at a dose of 400 mg/m2 of body surface area and then at 250 mg weekly during the whole RT.<br>**RT:** 70 Gy in 35 daily fractions, 5 days/week, over 7 weeks. It was delivered using 3D conformal RT. |
| **Rades 2023** | Retrospective cohort | CTX-RT | a total of 176 patients treated with chemoradiation for SCCHN between 2012 and 2022 were included in this retrospective study. | NA | **CB:** with AUC 1.0 on days 1 to 5 or AUC 1.5 on days 1 to 4<br>**RT:** planned doses of EBRT, administered as VMAT, were 60–70 Gy with doses per fraction of 2 Gy, given on 5 consecutive days per week. The doses were 60 Gy after microscopically complete resection, 66 Gy after microscopically incomplete resection and 70 Gy for definitive treatment. |
| **Rambeau 2017** | Retrospective cohort | CTX-RT | patients treated by cetuximab-radiotherapy, for a locally advanced head and neck squamous cell carcinoma (stage III and IV), with clinical contraindication to platinum-based chemotherapy, who received at least one cetuximab infusion. | administration of induction chemotherapy and previous neck irradiation. | **CTX:** weekly cetuximab initially at a dose of 400 mg/m2 of body surface area and then at 250 mg weekly during the whole RT.<br>**RT:** the planned total radiation dose was 70 Gy (2 Gy/day, 5 days/week) in invaded areas and 50-56 Gy in uninvolved areas, delivered using IMRT or 3D conformal radiotherapy |
| **Saigal 2014** | Retrospective cohort | CB-RT + CTX | patients treated with concurrent CCRT at the University of Miami Sylvester Comprehensive Cancer Center between 1/2007 and 12/2011. In total, 16 patients were identified who presented with newly diagnosed, biopsy-proven non-metastatic squamous cell carcinomas of the oropharynx, larynx, or hypopharynx who then completed a course of organ-preserving therapy with this regimen. | NA | **CTX:** weekly cetuximab initially at a dose of 400 mg/m2 of body surface area and then at 250 mg weekly during the whole RT, along with weekly carboplatin at an AUC of 1.5–2mg/ml/min.<br>**RT:** the planned dose was 70 Gy (2 Gy/day, 5 days/week) in gross disease in 33-35 fractions delivered using IMRT. |
| **Srinivas 2020** | Retrospective cohort | Nimo-RT | inclusion criteria for this retrospective study were as follows: (1) age 18 years and above, (2) tumor Stage III– IVb, (3) squamous histology, (4) Eastern Cooperative Oncology Group (ECOG) performance score ≤3, (5) unfit for surgery, (6) ineligible for chemotherapy (especially platinum-based), and (7) treated with injection nimotuzumab 200 mg IV weekly as infusion and concurrent intensity-modulated radiation therapy (IMRT) with total dose between 60 and 70 Gy. | exclusion criteria included nonsquamous histology and recurrent cancers with a history of prior chemoradiation and/or immunotherapy. | **Nimotuzumab:** 200 mg IV weekly as infusion and concurrent IMRT<br>**RT:** IMRT with a total dose between 60 and 70 Gy |
| **Sun 2022** | Retrospective cohort | CB-RT (N=1231)<br>CTX-RT (N=1493) | veterans who either initially received their diagnosis or sought their first treatment for cancer in the VA are recorded as complete analytic cases in the cancer registry. Complete HNSCC analytic cases diagnosed from 2006 to 2020 were identified, and patients identified in the cancer registry as receiving definitive CRT with cisplatin, carboplatin, or cetuximab were included in the study cohort. | patients with distant meta-static disease at diagnosis, patients who received radiotherapy or systemic therapy alone, and patients who underwent sur- gery as part of their primary treatment were excluded. | Most CB-treated patients also received treatment with paclitaxel; other CB partners included fluorouracil, capecitabine, and docetaxel. |

| | | | | | |
|---|---|---|---|---|---|
| **Swiecicki 2020** | phase II trial | CTX-RT | pathologically confirmed, previously untreated locally advanced squamous cell carcinoma of the larynx, hypopharynx, oropharynx, oral cavity, or unresectable head and neck cutaneous squamous cell carcinomas. Eligible patients were required to have met at least one of the following criteria 1) age ≥ 70 years old, 2) ECOG ≥ 1, 3) creatinine clearance < 30 cc/min, and/or 4) co-morbidities that precluded treatment with standard platinum-based chemotherapy as determined by the treating physician. | patients with nasopharyngeal squamous cell carcinoma were excluded. Patients with a history of head and neck radiation or chemotherapy, prior head and neck malignancy, previous treatment with anti-EGFR-directed therapy, or documented distant metastases were excluded. | **CTX:** weekly cetuximab initially at a dose of 400 mg/m2 of body surface area and then at 250 mg weekly during the whole RT.<br>**RT:** daily fractionated IMRT delivered over 35 fractions. For the cisplatin-ineligible cohort, a planning target volume mid to 63Gy was used for high-risk nodal volumes at the discretion of treating physicians. For the HPV low-risk cohort the planning target volume high was 70Gy and low was 56Gy where gross tumor volume was expanded by 3-5mm to create clinical target volume. |
| **Tao 2023** | Phase II trial | CTX-RT (N=66)<br>Pembro-RT (N=67) | age 18-80 years; Eastern Cooperative Oncology Group performance status (PS) of 0-1; patient unfit for receiving high-dose cisplatin-based chemotherapy; histologically confirmed non- metastatic SCCs of the oral cavity, oropharynx, hypopharynx, or larynx stage III-IV (American Joint Committee on Cancer Staging System 7th edition); known p16 status for oropharynx tumors; and the need for adequate organ function laboratory values, including absolute neutrophil count 1500/ml; platelets 100 000/ml; hemoglobin 9 g/ dl; serum creatinine 1.5 upper limit of normal (ULN); serum total bilirubin 1.5 ULN; aspartate aminotransferase and alanine aminotransferase 2.5 ULN. | (1) nasopharyngeal, paranasal sinuses, nasal cavity tumours or thyroid cancers; (2) squamous cell cancer involving cervical neck nodes with unknown primary site; (3) metastatic disease; (4) any prior or current treatment for invasive head and neck cancer; (5) weight loss of >10% during the last 3 weeks prior the screening visit; (6) concurrent treatment with any other systemic anti-cancer therapy that is not specified in the protocol; (7) concomitant treatment with any drug on the prohibited medication list such as live vaccines (for details, see the protocol); (8) history of another malignancy within the last 3 years (exception of in situ carcinoma and skin carcinomas). | **Pembro:** 200 mg given every 3 weeks**.**<br>**CTX:** 400 mg/m2 given day 8, followed by a weekly dose of 250 mg/m2 during RT.<br>**RT:** IMRT to the high-risk clinical target volume was delivered daily to a total dose of 69.96 Gy over 6.5 weeks (33 fractions of 2.12 Gy, 5 days/week). Elective areas with the low-risk clinical target volume received a total dose of 52.8 Gy (33 fractions of 1.6 Gy). |
| **Van Der Linden 2013** | Retrospective cohort | CTX-RT (N=61)<br>RT (N=80) | patients treated with first-line RT and CTX or RT for oropharyngeal, hypopharyngeal or laryngeal SCC between January 1, 2007 and December 31, 2010 in two university medical centers. No further selection criteria were applied. | NA | **RT: r**egimens used in daily practice were diverse. Preferred regimen was accelerated RT, 35 9 2 Gy, total dose 70 Gy, 6 fractions per week. |
| **Ueki 2023** | Phase II trial | CB-RT | 20 ≤ age ≤ 90; histologically proven HNSCC; treated with radical chemoradiotherapy, i.e., whole-neck irradiation including bilateral levels II–IV; measurable lesion according to Response Evaluation Criteria in Solid Tumors (RECIST) version 1.1; patients with renal dysfunction (30–60 mL/min of estimated glomerular filtration rate aged less than 75 years old; ≥ 75-year-old patients with 40 mL/min of eGFR or better; ECOG of 0 to 2; adequate organ functions 14 days prior to registration. Patients were required to meet the following laboratory criteria: white blood cells > 3000 /mm3; neutrophils > 1500 /mm3; platelets > 100,000 /mm3; and total bilirubin, aspartate aminotransferase (AST), and alanine aminotransferase (ALT) less than two times the upper normal limit. | NA | **CB:** administered at AUC 2 weekly for seven cycles during the RT period. It was administered at a reduced dose (AUC = 1–1.5) in cases of grade 2 hematological adverse events, and suspended when grade 3 or higher hematological toxicity occurred or when investigators judged it as inappropriate.<br>**RT:** administered with high-energy photons of 4–10 MV x-rays at a total dose of 70 Gy in 35 fractions over 7 weeks. 3D conformal radiation therapy or IMRT was chosen at the institutional discretion. |

| | | | | | |
|---|---|---|---|---|---|
| **Weiss 2020** | Phase II trial | Pembro-RT | patient were required to have untreated stage III-IV (non-metastatic) HNSCC, and a contraindication to cisplatin as defined by: abnormal renal function (GFR< institutional lower limit of institutional normal (<LLN)); abnormal hearing (patient or audiology defined); pre-existing tinnitus; neuropathy (bilateral paresthesias or loss of deep tendon reflexes in upper and/or lower extremities); diabetes mellitus; oncologist-certification that patient would not be considered eligible for high dose cisplatin when given as standard of care (for example, due to age or another medical problem, reason was required to be documented); patient refusal for high dose cisplatin. Patients were required to have ECOG PS 0–1 and normal organ function. | nasopharyngeal carcinoma was excluded, as well as active autoimmune disease, a requirement for immunomodulating medications, active infection or non-infectious pneumonitis. | **Pembro:** 200mg IV concurrent with radiation, at time points 0, 3 weeks and 6 weeks and for three adjuvant cycles following.<br>**RT:** 70Gy of IMRT in 35 fractions |
| **Ye 2013** | Retrospective cohort | CTX-RT | patients with stage III and IV HNSCC treated with concomitant therapy between August 1, 2007 and July 31, 2010. This time period corresponds with the first approval of the use of CTX with RT in HNSCC. | previous head and neck malignancies, primary treatment with surgery, induction chemotherapy or nasopharyngeal primaries. Electronic charts, including RT treatment plans, were reviewed in 262 RT-CIS and 87 RT-CET cases to collect data on treatment, toxicity, response, and outcomes. | **CTX:** weekly cetuximab initially at a dose of 400 mg/m2 of body surface area and then at 250 mg weekly during the whole RT.<br>**RT:** standard (70 Gy in 2 Gy daily fractions over 7 weeks), hypofractionated (60 Gy in 2.4 Gy daily fractions over 5 weeks) or concomitant boost (66-70 Gy in 2 Gy fractions with additional second daily fraction once per week separated by greater than 6 h over 5.5-6 weeks) RT. High dose (60-70 Gy) was delivered to the primary and involved nodes, intermediate dose (50-56 Gy) to the uninvolved neck. Most treatment was with IMRT and some with 3D conformal RT. |

'NA' was reserved for studies lacking an explicit description of exclusion criteria. AUC: area under the curve; CB: carboplatin; CET: cetuximab CTX: cetuximab; Cis: cisplatin; DTX: docetaxel; EBRT: external beam radiotherapy; ECOG PS: Eastern Cooperative Oncology Group Perfomance Status; HNSCC: head and neck squamous cell carcinoma; HNC: head and neck cancer; IMRT: intensity modulation radiotherapy; NA: not available; N: Number of patients; Nimo: nimotuzumab; PTX: paclitaxel; Pemb: Pembrolizumab; Pembro: pembrolizumab; RT: radiotherapy; 3D: three-dimensional; VMAT: Volumetric modulated arc therapy.

**Table S4: Definition of outcomes according to the information available in studies included in this systematic review.**

| Study | Overall survival (OS) | Progression-free survival (PFS) | Disease-free survival (DFS) | Locoregional control (LRC) | Recurrence | Other |
|---|---|---|---|---|---|---|
| **Addeo 2019** | period from the first infusion of cetuximab until death from any cause | period from the first infusion of cetuximab until the first record of disease progression | NA | absence of progression of loco-regional disease at the time of scheduled follow-up visits | NA | NA |
| **Agarwal 2011** | NA | NA | NA | NA | recurrence was defined as reappearance of disease at either the primary, regional or distant site | locoregional failure (LRF): persistence of disease or reappearance of disease either at the primary site and/or draining regional lymph nodes |
| **Beckham 2019** | NA | NA | NA | NA | recurrence outside of the head and neck | LRF: recurrence at the site of initial primary or cervical lymph nodes<br>distant metastatic recurrence: recurrence outside of the head and neck |
| **Corry 2017** | NA | NA | NA | NA | NA | NA |
| **Fung 2020** | the time from diagnosis to death from any cause | NA | time from the end of treatment to recurrence or death | NA | NA | NA |
| **Hamauchi 2019** | calculated from the first day of RT until death from any cause or censored at the last follow-up visit | calculated from the first day of RT until disease relapse, PD, death from any cause, or cen- sored at the last follow-up visit | NA | NA | NA | NA |
| **Han 2023** | death from any cause | any local, regional, or distant recurrence or death from any cause | NA | NA | locoregional recurrence: any local or regional recurrence | distant metastatic recurrence: any distant spread of disease |
| **Imai 2022** | calculated from the first day of treatment to death or to censoring for any reason | NA | NA | defined as no progression of local disease during the follow-up period | NA | NA |

| | | | | | | |
|---|---|---|---|---|---|---|
| **Magnes 2021** | time between primary diagnosis and death from any cause | date of primary diagnosis until disease progression or death from any cause | NA | NA | NA | NA |
| **Maring 2018** | end of radiotherapy until date of death | NA | NA | NA | NA | event-free survival (EFS): initiation of RT until date of documented recurrence or death |
| **Nassif 2022** | NA | NA | NA | NA | NA | NA |
| **Patil 2023** | time in years between the date of random assignment and the date of death | NA | time in years between the date of random assignment and the date of the first episode of disease recurrence or progression or death because of any cause, whichever occurred earlier | NA | NA | locoregional failure-free survival (LRFS) was defined as the time in years between the date of random assignment and the date of the first episode of locoregional disease recurrence or progression or death because of any cause, whichever occurred earlier |
| **Pryor 2009** | NA | NA | NA | NA | NA | NA |
| **Rades 2023** | NA | NA | NA | NA | NA | NA |
| **Rambeau 2017** | NA | NA | NA | NA | NA | NA |
| **Saigal 2014** | time from diagnosis to death from any cause | The date of progression was selected as the date of the first event including locoregional recurrence (LRR), distant metastasis, or death | NA | NA | Locoregional recurrence (LRR): recurrence at the primary site or in the neck after a disease-free interval | NA |
| **Srinivas 2020** | NA | NA | NA | NA | NA | NA |
| **Sun 2022** | time from treatment initiation to death of any cause | NA | NA | NA | NA | NA |
| **Swiecicki 2020** | time from enrollment to death from any cause, or date of last follow-up for alive patients | NA | time from enrollment to first event of either recurrent disease or death | NA | NA | freedom from local regional progression (FFLRP): time from enrollment to first local or regional failure |
| **Tao 2023** | time to death from any cause measured from randomization | time from randomization to progression/relapse at any site (local, regional, or distant) or to death from any cause | NA | absence of disease progression or recurrence at the site of the primary tumor and locoregional lymph nodes | NA | NA |
| **Van Der Linden 2013** | time from treatment start to death | NA | NA | NA | NA | NA |
| **Ueki 2023** | NA | NA | NA | NA | NA | NA |
| **Weiss 2020** | time from the start of treatment to death from any cause | start of treatment to the first occurrence of progressive disease (RECIST or clinically defined) or death as a first event | NA | NA | NA | NA |
| **Ye 2013** | DFS and OS were measured from the beginning of RT to the | NA | DFS included locoregional relapses and distant metastases but did not include second malignancies | beginning of RT to primary or neck relapse and included untreated persistent disease | NA | NA |

| | | | | | | |
|---|---|---|---|---|---|---|
| | date of documented disease,<br>last follow-up or death | | | | | |

NA: study does not assess the specific outcome or the outcome definition was not available.

**Table S5: Other outcomes not included in main tables.**

| Study | Overall survival (OS) | Progression-free survival | Locoregional control (LRC) | Disease-free survival (DFS) | Recurrence | Other outcomes |
|---|---|---|---|---|---|---|
| **Addeo 2019** | **-Median OS by site:**<br>Oropharynx: 24 (95% CI 23.4–24.6)<br>Larynx: 40 (95% CI 37.8–42.2)<br>Hypopharynx: 30 (95% CI 28.4–31.5) | NA | **-Median LRC by site:**<br>Oropharynx: 14.6 (12.9–15.3)<br>Larynx: 19.2 (17.9–19.9)<br>Hypopharynx: 16.7 (15.7–17.5) | NA | NA | NA |
| **Agarwal 2011** | **-2-year OS in oropharyngeal vs non-oropharyngeal:**<br>54.8% vs 33.4%<br>**-2-year OS node negative versus positive:**<br>45.2% X 44.6% | NA | **-2-year LRC in oropharyngeal X non-oropharyngeal:**<br>44.4% X 27.1%<br>**-2-year LRC node negative versus positive:**<br>43.8% X 28.3% | **-2-year DFS in oropharyngeal X non-oropharyngeal:**<br>33.3% X 22.7%<br>**-2-year DFS node negative versus positive:**<br>35% X 26.7% | NA | NA |
| **Beckham 2019** | **3-year OS (HR; 95% CI):**<br>CB-RT: 0.59 (95% CI 0.47–0.72)<br>CTX-RT 0.54 (95% CI 0.40–0.68)<br><br>**Propensity score-matched analysis (HR, 95% CI) for OS** in patients treated with Cis-RT, CB-RT and CTX-RT showed better outcomes for Cis-RT and CB-RT groups:<br>0.56 (95% CI 0.31–0.99; p = 0.05) | NA | NA | NA | **3-year distant metastasis incidence (HR; 95% CI):**<br>CB-CRT 0.12 (95% CI 0.04–0.20)<br>CTX-RT: 0.25 (95% CI 0.13–0.37)<br><br>**Multivariable analysis:**<br>**LRF hypopharynx vs larynx and oroharynx (HR, 95% CI):** 0.64 (95% CI 0.39 -1.02, p=0.06)<br>**Distant metastasis:** 0.82 (0.47 - 1.43, p=0.48)<br><br>**Propensity score-matched analysis (HR, 95% CI) for LRF** in patients treated with Cis-RT, CB-RT and CTX-RT showed better outcomes for Cis-RT and CB-RT groups: 0.38 (95% CI 0.17–0.85; p = 0.02) | **3-year LRF (HR; 95% CI):**<br>CB-RT: 0.18 (95% CI 0.09–0.28)<br>CTX-RT: 0.48 (95% CI 0.34–0.62) |

| | | | | | | |
|---|---|---|---|---|---|---|
| **Corry 2017** | **-4-year OS in HPV+ vs HPV-**<br>93% (95% CI: 84-100) vs 50% (95% CI: 22-100) | NA | NA | NA | NA | NA |
| **Fung 2020** | -**Median OS in HPV+ vs HPV- patients:** 27 vs 44 (HR 5.37 [95% CI NA]) | NA | NA | **-Median DFS:** 52 (IQR 29.0-78)<br>-**Median DFS in HPV+ vs HPV- patients:** 15 vs 38; (HR 4.22 [95% CI NA]) | **-Locoregional recurrence:**<br>CTX-RT: 10 (34.5)<br>**-Distant:**<br>CTX-RT: 8 (27.6)<br>**-Both:**<br>CTX-RT: 11 (37.9) | NA |
| **Hamauchi 2019** | **-OS in CB-RT vs CTX-RT (HR, 95% CI):** HR 0.69 (95% CI 0.63–3.37; p = 0.38) | **-OS in CB-RT vs CTX-RT:** HR: 0.74 (95% CI 0.63–2.89; p = 0.44) | **LRC in CB-RT vs CTX-RT:** (HR 1.17; 95% CI 0.34-2.15; p=0.74) | NA | NA | NA |
| **Han 2023** | **-OS in oropharyngeal p16+ vs p16- (HR, 95% CI):** HR 0.55 (95% CI 0.10–3.04; p = 0.5)<br>**-OS in oropharyngeal p16+ vs all other patients (HR, 95% CI):** HR 0.58 (95% CI 0.18-1.84; p = 0.4)<br>**-OS in N2/N3 vs N0/N1 (HR, 95% CI):** HR: 1.06 (95% CI 0.28–4.0; p = 0.9) | NA | NA | NA | NA | NA |
| **Imai 2022** | **-Multivariable analysis for OS in oropharynx vs larynx/hypopharynx (HR, 95% CI):** 1.94 (95% CI NA, p=0.12) | NA | NA | NA | NA | NA |
| **Magnes 2021** | **-OS in CTX-RT vs RT (HR, 95% CI):** HR: 0.68 (95% CI: 0.34–1.35; p = 0.26)<br>-Age, performance score, AJCC stage and resection margins had no statistically significant influence on PFS and OS of patients | **-PFS of CTX-RT vs RT alone (HR, 95% CI):** HR: 0.98 (95% CI 0.53–1.82; p = 0.96) | NA | NA | NA | NA |
| **Maring 2018** | NA | NA | NA | NA | NA | NA |
| **Nassif 2022** | NA | NA | NA | NA | NA | NA |

| | | | | | | |
|---|---|---|---|---|---|---|
| **Patil 2023** | **-OS in RT vs RT-DTX according to tumor location (HR, 95% CI):**<br>**Hypolarynx:** 0.45 (95% CI 0.22 - 0.92, p=0.29)<br>**Larynx:** 0.75 (95% CI 0.35 - 1.64, p=0.483)<br>**Oral cavity:** 0.88 (95% CI 0.55 - 1.4, p=0.594)<br>**Oropharynx:** 0.86 (95% CI 0.54 - 1.37, p=0.552). | NA | NA | **-DFS in RT vs RT-DTX according to tumor location (HR, 95% CI):**<br>**Hypolarynx:** 0.33 (95% CI 0.16 - 0.65, p=0.001)<br>**Larynx:** 0.74 (95% CI 0.37 to 1.47, p=0.393)<br>**Oral cavity:** 0.83 (95% CI 0.53 to 1.3, p=0.423)<br>**Oropharynx:** 0.83 (95% CI 0.54 to 1.27, p=0.405) | NA | **Median locoregional failure free survival (95% CI):**<br>RT: 5.9 (95% CI, 4.9 to 7.5)<br>DTX-RT: 12.4 (95% CI, 8.6 to 23.5) |
| **Pryor 2009** | NA | NA | NA | NA | NA | NA |
| **Rades 2023** | NA | NA | **Multivariable analysis of LRC considering HPV status (positive vs negative) [HR, 95% CI]:** 3.46 (95% CI 1.02-11.76; p = 0.047) | NA | NA | **Metastases-free survival (MFS) in the CB-RT:**<br>1-year MFS: 87%<br>2-year MFS: 78%<br>3-year: 74% |
| **Rambeau 2017** | **Median OS (95% CI):** 12.8 (95% CI 8.7-20.2) | **Median PFS (95% CI):** 6.5 (95% CI 6-10.1) | NA | NA | NA | NA |
| **Saigal 2014** | NA | NA | NA | NA | NA | NA |
| **Srinivas 2020** | NA | NA | NA | NA | NA | NA |
| **Sun 2022** | **-Median OS (95% CI):**<br>CB-RT: 43.4 (15.3-123.8)<br>CTX-RT: 31.1 (12.4-87.8)<br><br>**-Median OS (95% CI) and 5-year OS in CB-RT:**<br>**-Oropharyngeal cancer (OPC):** 69.5 (NA) and 5-year survival, 53.0% (95% CI, 49.1%-56.8%)<br>**-Non-OPC**:30.9 (NA) and 5-year survival, 31.8% (95% CI, 27.9%-35.8%)<br><br>**-Median OS (95% CI) and 5-year OS in CTX-RT:**<br>**-OPC:** 44.1 (NA); 5-year survival, 42.2% (95% CI, 38.8%-45.6%)<br>**-Non-OPC:**, 20.9 (NA); 5-year survival, 24.7% (95% CI, 21.2%-28.2%)<br><br>-CB was associated with improved survival compared with CTX in the oropharynx subgroup (csHR, 0.82; 95% CI, 0.72-0.94); in the non-oropharynx subgroup, survival with treatment with CB was numerically but not statistically superior to CTX (csHR, 0.88; 95% CI, 0.78-1.00) | NA | NA | NA | NA | NA |
| **Swiecicki 2020** | NA | NA | NA | NA | NA | **FFRLP:** 14/21 patients (1 year FFLRP); 11/21 (2 year FFLRP) |

| | | | | | | |
|---|---|---|---|---|---|---|
| **Tao 2023** | **Adjusted HR (95% CI) for OS in RT vs Pembro--CTX:** 0.73 (0.42-1.26), p=0.25<br><br>The reference arm was the cetuximabeRT arm; adjustment for stratification factors (node involvement and p16 status) and stage (III/IVa/IVb). | **Adjusted HR (95% CI) for PFS in RT vs Pembro--CTX**: 0.73 (0.46-1.16), p=0.18<br><br>The reference arm was the cetuximab-RT arm; adjustment for stratification factors (node involvement and p16 status) and stage (III/IVa/IVb). | **Adjusted HR (95% CI) for LRC in RT vs Pembro--CTX:** 1.42 (0.54-3.75), p=0.48<br><br>The reference arm was the cetuximab-RT arm; adjustment for stratification factors (node involvement and p16 status) and stage (III/IVa/IVb). | NA | NA | NA |
| **Van Der Linden 2013** | NA | NA | NA | NA | NA | NA |
| **Ueki 2023** | NA | NA | NA | NA | NA | NA |
| **Weiss 2020** | **1-year OS in p16 associated oropharynx cancer:** 93% (59–99%)<br>**1-year OS in non-oroparhynx plus p16−oropharynx:** 80% (50–93%) | **1-year PFS in p16 associated oropharynx cancer**: 86% (54–96%)<br>**1-year OS in non-oroparhynx plus p16−oropharynx:** 67% (38%-85%) | NA | NA | NA | NA |
| **Ye 2013** | **OS in oropharynx vs non-oropharynx (HR, 95% CI):**<br>1.0 (NA) vs 3.16 (NA), p<0.01 | NA | **LRC in oropharynx vs non-oropharynx (HR, 95% CI):**<br>1.0 (NA) vs 5.09 (NA), p<0.01 | **DFS in oropharynx vs non-oropharynx (HR, 95% CI):**<br>1.0 (NA) vs 3.10 (NA), p<0.01 | NA | NA |

NA: study does not assess the specific outcome or outcome already included in a pooled analysis; 95% CI: confidence interval; Median values and time-related outcomes are given in months followed by range in parenthesis unless indicated otherwise. DRFS: distant recurrence-free survival; EFS: event-free survival; FFLRP: Freedom from local regional progression; HR: Hazard ratio; HPV: human papillomavirus; (+) positive; (-) negative; IQR: interquartile range; LRF: locoregional failure; LRFS: locoregional recurrence-free survival; OPC: oropharyngeal carcinoma; RFS: recurrence-free survival.

**Table S6: Detailed information regarding tumor staging based on available information of studies included in this systematic review.**

| Study | Design | Treatment regimen | N | Disease stage | | | Tumor stage (N) | | | | | | Nodal stage (N) | | | | | Staging system |
|---|---|---|---|---|---|---|---|---|---|---|---|---|---|---|---|---|---|---|
| | | | | I/II | III | IV | TX | T0 | T1 | T2 | T3 | T4 | Nx | NO | N1 | N2 | N3 | |
| **Addeo 2019** | Retrospective cohort | CTX-RT | 64 | 0 | 25 (39.1%) | 39 (60.1%) | NA | NA | NA | NA | NA | NA | NA | NA | NA | NA | NA | NA |
| **Agarwal 2011** | Retrospective cohort | CTX-RT | 37 | 3 (8.1%) | 13 (35.1%) | 21 (56.8%) | 1 (2.7%) | 0 | 0 | 11 (29.7%) | 14 (37.8%) | 11 (29.72%) | 0 | 16 (43.2%) | 8 (21.6%) | N2a: 3 (8.1%)<br>N2b: 10 (27.02%) | 0 | AJCC 6th ed |
| **Beckham 2019** | Retrospective cohort | CB-RT (N=74)<br>CTX-RT (N=61) | 135 | 0 | CB-RT: 57 (77%)<br>CTX-RT: 49 (80.3%) | CB-RT: 17 (23%)<br>CTX-RT: 12 (19.7%) | 0 | 0 | CB-RT: 5 (6.8%)<br>CTX-RT: 3 (4.9%) | CB-RT: 19 (25.7%)<br>CTX-RT: 15 (24.9%) | CB-RT: 33 (44.6%)<br>CTX-RT: 31 (50.8%) | CB-RT: 17 (23%)<br>CTX-RT: 12 (19.7%) | 0 | CB-RT: 15 (20.3%)<br>CTX-RT: 18 (29.5%) | CB-RT: 13 (17.6%)<br>CTX-RT: 17 (27.9%) | CB-RT: 45 (60.8%)<br>CTX-RT: 26 (42.6%) | CB-RT: 1 (1.4%)<br>CTX-RT: 2 (1.1%) | AJCC 7th ed |
| **Corry 2017** | Phase I/II trial | CB-RT + CTX | 60 | 0 | 14 (23%) | 46 (77%) | 0 | 0 | 3 (5%) | 24 (40%) | 26 (43%) | 7 (12%) | 0 | 12 (20%) | 7 (12%) | 2a: 5 (8%)<br>2b: 21 (35%)<br>2c: 12 (20%) | 3 (5%) | NA |
| **Fung 2020** | Retrospective cohort | CTX-RT | 115 | 0 | 12 (10.4%) | 103 (89.6%) | 0 | 0 | 29 (52.2%) | 37 (32.2%) | 18 (15.7%) | 31 (27%) | 0 | 8 (7%) | 12 (10.4%) | 91 (79.1%) | 4 (3.5%) | AJCC 6th ed |
| **Hamauchi 2019** | Retrospective cohort | CB-RT (N=29)<br>CTX-RT (N=18) | 47 | 0 | CB-RT: 3 (10.3%)<br>CTX-RT: 9 (50%) | CB-RT: 26 (89.7%)<br>CTX-RT: 9 (50%) | CB-RT: 0<br>CTX-RT: 0 | CB-RT: 0<br>CTX-RT: 0 | CB-RT: 2 (7%)<br>CTX-RT: 0 | CB-RT: 8 (27.6%)<br>CTX-RT: 5 (27.8%) | CB-RT: 5 (27.6%)<br>CTX-RT: 10 (55.5%) | CB-RT: 14 (48.3%)<br>CTX-RT: 3 (16.7%) | 0 | CB-RT: 5 (17.2%)<br>CTX-RT: 6 (33.3%) | CB-RT: 1 (3.5%)<br>CTX-RT: 4 (22.2%) | CB-RT: 22 (75.9%)<br>CTX-RT: 8 (44.4%) | CB-RT: 1 (3.5%)<br>CTX-RT: 0 | AJCC 7th ed |
| **Han 2023** | Retrospective cohort | CB-RT + paclitaxel | 65 | NA | NA | NA | 0 | 2 (3.1%) | 6 (9.2%) | 24 (36.9%) | 20 (30.8%) | 13 (20%) | 0 | 9 (13.8%) | 9 (13.8%) | 46 (70.8%) | 1 (1.5%) | NA |
| **Imai 2022** | Retrospective cohort | CTX-RT | 88 | 0 | 17 (19.3%) | 71 (80.7%) | 0 | 0 | 3 (3%) | 32 (36%) | 26 (30%) | 27 (31%) | 0 | 20 (23%) | 6 (7%) | 59 (67%) | 3 (3%) | NA |
| **Magnes 2021** | Retrospective cohort | CT-RT (N=26)<br>RT (N=56) | 82 | CTX-RT: 1 (3.8%)<br>RT: 10 (17.8%) | CTX-RT: 3 (11.5%)<br>RT: 16 (28.6%) | CTX-RT: 18 (69.2%)<br>RT: 24 (42.9%) | NA | NA | NA | NA | NA | NA | NA | NA | NA | NA | NA | AJCC 7th ed |
| **Maring 2018** | Retrospective cohort | CB-RT + paclitaxel | 23 | 0 | 4 (17%) | 19 (83%) | 0 | 0 | 0 | 6 (26%) | 10 (44%) | 7 (30%) | NA | NA | NA | NA | NA | AJCC 7th ed |
| **Nassif 2022** | Retrospective cohort | CB-RT + paclitaxel | 30 | 0 | 5 (16.7%) | 25 (83.3%) | 0 | 2 (6.7%) | 3 (10%)[a] | a | 25 (83.3%)[a] | a | 0 | 7 (23.3%) [a] | a | 23 (76.6%)[∝] | a | AJCC 7th or 8th ed |
| **Patil 2023** | Phase II/III trial | RT (N=176)<br>DTX-RT (N=180) | 356 | 0 | RT: 34 (19.3%)<br>DTX-RT: 43 (23.9%) | RT: 142 (80.7%)<br>DTX-RT: 137 (76.1%) | 0 | RT: 6 (3.4%)<br>DTX-RT: 4 (2.2%) | RT: 11 (6.2%)<br>DTX-RT: 9 (5%) | RT: 23 (13.1%)<br>DTX-RT: 26 (14.4%) | RT: 51 (29%)<br>DTX-RT: 52 (28.9%) | RT: 85 (48.3%)<br>DTX-RT: 89 (49.5%) | 0 | RT: 26 (14.8%)<br>DTX-RT: 38 (21.1%) | RT: 42 (23.9%)<br>DTX-RT: 35 (19.4%) | RT: 93 (52.8%)<br>DTX-RT: 93 (51.7%) | RT: 15 (8.5%)<br>DTX-RT: 14 (7.8%) | AJCC 7th ed |

| | | | | | | | | | | | | | | | | | | |
|---|---|---|---|---|---|---|---|---|---|---|---|---|---|---|---|---|---|---|
| **Pryor 2009** | Prospective cohort | CTX-RT | 13 | 0 | 4 (31%) | 9 (69%) | 0 | 0 | 2 (15.4%) | 2 (15.4%) | 5 (38.5%) | 3 (18.8%) | 0 | 4 (30.8%) | 2 (15.4%) | 6 (46.2%) | 0 | NA |
| **Rades 2023** | Retrospective cohort | CTX-RT | 45 | NA | NA | NA | 0 | 0 | 12 (27%)[b] | b | 12 (27%) | 21 (47%) | 0 | 15 (33%)[b] | b | b | 30 (67%)[b] | NA |
| **Rambeau 2017** | Retrospective cohort | CTX-RT | 88 | 0 | 22 (25%) | 62 (70.4%) | NA | NA | NA | NA | NA | NA | NA | NA | NA | NA | NA | NA |
| **Saigal 2014** | Retrospective cohort | CTX-RT + CTX | 16 | 1 (6.3%) | 10 (62.5%) | 5 (31.2%) | 1 (6.3%) | 0 | 1 (6.3%) | 5 (31.2%) | 8 (50%) | 1 (6.3%) | 0 | 8 (50%) | 5 (31.2%) | 3 (18.8%) | 0 | AJCC 7th ed |
| **Srinivas 2020** | Retrospective cohort | Nimo-RT | 21 | 0 | 7 (33.3) | 14 (66.7%) | 0 | 0 | 0 | 4 (19%) | 7 (33.3%) | 10 (47.6%) | 0 | 0 | 4 (19%) | 13 (61.9%) | 4 (19%) | NA |
| **Sun 2022** | Retrospective cohort | CB-RT (N=1231) CTX-RT(N=1493) | 2724 | NA | NA | NA | 0 | CB-RT: 30 (2.5%) CTX-RT: 18 (1.3%) | CB-RT:134 (11.4%) CTX-RT: 167 (11.9%) | CB-RT: 417 (35.4%) CTX-RT: 437 (31.1%) | CB-RT: 320 (27.2%) CTX-RT: 437 (31.1%) | CB-RT: 277 (23.5%) CTX-RT: 344 (24.5%) | 0 | CB-RT: 264 (22.4%) CTX-RT: 306 (21.8%) | CB-RT: 178 (15.1%) CTX-RT: 219 (15.6%) | CB-RT: 679 (57.7%) CTX-RT: 807 (57.5%) | CB-RT: 56 (4.8%) CTX-RT: 71 (5.1%) | NA |
| **Swiecicki 2020** | Phase II trial | CTX-RT | 21 | 0 | 6 (28.6%) | 15 (71.4%) | 0 | 1 (5%) | 8 (38%)[c] | c | 6 (29%) | 6 (29%) | 0 | 8 (38%)[c] | c | 9 (43%)[c] | 4 (19%)[c] | AJCC 7th ed |
| **Tao 2023** | Phase II trial | CTX-RT (N=66) Pembro-RT (N=67) | 133 | 0 | CTX-RT: 17 (26%) Pembro-RT: 16 (24%) | CTX-RT: 48 (74%) Pembro-RT: 50 (76%) | NA | NA | NA | NA | NA | NA | NA | NA | NA | NA | NA | AJCC 7th ed |
| **Van Der Linden 2013** | Retrospective cohort | CTX-RT (N=61) RT (N=80) | 141 | 0 | CTX-RT: 16 (26.2%) RT: 35 (43.7%) | CTX-RT: 45 (73.8%) RT: 45 (56.2%) | NA | NA | NA | NA | NA | NA | NA | NA | NA | NA | NA | NA |
| **Ueki 2023** | Phase II trial | CB-RT | 30 | 7 (14.3%) | 10 (33.3%) | 13 (43.3%) | NA | NA | NA | NA | NA | NA | NA | NA | NA | NA | NA | NA |
| **Weiss 2020** | Phase II trial | Pembro-RT | 29 | 8 (27.6%) | 11 (37.9%) | 10 (34.5%) | NA | NA | NA | NA | NA | NA | NA | NA | NA | NA | NA | AJCC 7th or 8th ed |
| **Ye 2013** | Retrospective cohort | CTX-RT | 87 | 0 | 27 (31%) | 60 (69%) | NA | NA | NA | NA | NA | NA | NA | NA | NA | NA | NA | NA |

Data is given as numbers unless indicated otherwise; AJCC: American Joint Committee on Cancer; CB: carboplatin; CTX: cetuximab; DTX: docetaxel; ECOG PS: Eastern Cooperative Oncology Group Perfomance Status; Ed: edition; NA: not available or subgroup not assessed by the study; N: Number of patients; Nimo: nimotuzumab; PTX: paclitaxel; Pembro: Pembrolizumab; RT: radiotherapy; a: In Nassif 2022 T stage was given as T1-T2 (3 patients) and T3/T4 (25 patients), N stage was given as N0/1 (7 patients) and N2/N3 (23 patients); b: In Rades 2023 T stage was given as T1-T2 (12 patients), and N stage as N0-N2a (15 patients) and N2b-N3 (30 patients); c: In Swiecicki 2021 T stage was given as T1-T2 (8 patients), N stage as N0-N1 (8 patients), N2a-N2b (9 patients) and N2c-N3c (4 patients).

**Table S7: List of excluded studies after a comprehensive review.**

| Author, year | Title | Exclusion reason |
|---|---|---|
| Okada 2023 | Effects of Pembrolizumab in Recurrent/Metastatic Squamous Cell Head and Neck Carcinoma: A Multicenter Retrospective Study | recurrent HNSCC; lack of population of interest |
| Maulik 2023 | Definitive chemoradiation for oropharyngeal squamous carcinomas: Outcomes with intensity-modulated radiation therapy using simultaneous integrated boost, in a majorly p16 negative cohort | lack of population of interest |
| Gillison 2023 | Safety of Nivolumab Added to Chemoradiation Therapy Platforms for Intermediate and High-Risk Locoregionally Advanced Head and Neck Squamous Cell Carcinoma: RTOG Foundation 3504 | only safety outcomes; lack of population of interest |
| Gillison 2019 | Safety and disease control achieved with the addition of nivolumab (Nivo) to chemoradiotherapy (CRT) for intermediate (IR) and high-risk (HR) local-regionally advanced head and neck squamous cell carcinoma (HNSCC): RTOG Foundation 3504 | only safety outcomes; lack of population of interest |
| Zděblová Čermáková 2023 | HPV and RAD51 as Prognostic Factors for Survival in Inoperable Oral and Oropharyngeal Cancer in Patients Unfit for Chemotherapy Treated with Hyperfractionated Radiotherapy | study does not specify if patients are cisplatin-unfit |
| Dechaphunkul 2018 | Comparison of 3-weekly cisplatin versus 3-weekly carboplatin in patients with locally advanced nasopharyngeal carcinoma (LA-NPC) receiving concurrent chemoradiotherapy (CCRT): A multicenter retrospective study | population overlap; another publication from the same study already assessed |
| Marques 2023 | Postoperative radiotherapy with cetuximab for head and neck squamous cell carcinoma patients at high risk of recurrence not eligible for cisplatin: A single-center experience and literature review | not in English |
| Alamgeer 2022 | Treatment outcomes of standard (high dose) cisplatin and non-standard chemotherapy for locally advanced head and neck cancer | lack of population of interest; all patients treated with cisplatin (high versus low dose) |
| Sun 2018 | A phase II randomized trial of pembrolizumab versus cetuximab, concomitant with radiotherapy (RT) in locally advanced (LA) squamous cell carcinoma of the head and neck (SCCHN): First results of the GORTEC 2015-01 “PembroRad” trial | population overlap; another publication from the same study already assessed |
| Bourhis 2020 | Pembrolizumab versus cetuximab, concomitant with radiotherapy (RT) in locally advanced head and neck squamous cell carcinoma (LA-HNSCC): Results of the GORTEC 2015-01 “PembroRad” randomized trial | population overlap; another publication from the same study already assessed |
| Morse 2023 | Treatment Tolerance of Cetuximab versus Alternative Chemotherapy Agents in Non-Cisplatin Candidates with Head and Neck Cancer Receiving Concurrent Chemoradiotherapy | only safety outcomes; lack of outcomes of interest |
| Dechaphunkul 2022 | Real-world evidence of cisplatin versus carboplatin in patients with locally advanced nasopharyngeal carcinoma receiving concurrent chemoradiotherapy: A multicenter analysis | lack of population of interest |
| Zandberg 2018 | Definitive radiation with concurrent cetuximab vs. radiation with or without concurrent cytotoxic chemotherapy in older patients with squamous cell carcinoma of the head and neck: Analysis of the SEER-medicare linked database | lack of population of interest |
| Xiang 2019 | Survival After Definitive Chemoradiotherapy With Concurrent Cisplatin or Carboplatin for Head and Neck Cancer | population overlap with other studies conducted in the same country |
| Ruhle 2023 | Evaluation of Concomitant Systemic Treatment in Older Adults With Head and Neck Squamous Cell Carcinoma Undergoing Definitive Radiotherapy | lack of data for the cisplatin-unfit patients |
| Hobday 2022 | Outcomes Among Patients With Mucosal Head and Neck Squamous Cell Carcinoma Treated With Checkpoint Inhibitors | non-LA HNSCC; lack of population of interest |
| Machiels 2015 | Afatinib versus methotrexate as second-line treatment in patients with recurrent or metastatic squamous-cell carcinoma of the head and neck progressing on or after platinum-based therapy (LUX-Head & Neck 1): an open-label, randomised phase 3 trial | includes only patients who progressed after cisplatin; recurrent HNSCC; lack of population of interest |
| Le Roy 2022 | Efficacy and Tolerance of Carboplatin plus Cetuximab (Simplified EXTREME Regimen) in Patients with Recurrent and/or Metastatic Head and Neck Squamous Cell Carcinoma | only safety outcomes; recurrent HNSCC; lack of population and outcomes of interest |
| Mell 2022 | Radiotherapy with Durvalumab vs. Cetuximab in Patients with Locoregionally Advanced Head and Neck Cancer and a Contraindication to Cisplatin: Phase II Results of NRG-HN004 | abstract only |
| Mell 2019 | Safety of radiotherapy with concurrent and adjuvant MEDI4736 (durvalumab) in patients with locoregionally advanced head and neck cancer with a contraindication to cisplatin: NRG-HN004 | population overlap; only safety data; lack of outcomes of interest |

| | | |
|---|---|---|
| NCT05784012 | Two-cohort Study of Niraparib and Dostarlimab Plus (Chemo)Radiotherapy in Locally-Advanced Head and Neck Squamous Cell Carcinoma (RADIAN) | active study, no results available |
| Ni W 2023 | Treatment Outcomes of Concurrent Nimotuzumab with Intensity Modulated Radiotherapy in Advanced Nasopharyngeal Carcinoma Patients Unfit for Concurrent Chemoradiotherapy: A Single Institute Experience | abstract only |
| Yom 2022 | NANORAY-312: A Phase III Pivotal Study of NBTXR3 Activated by Investigator's Choice of Radiotherapy Alone or Radiotherapy in Combination with Cetuximab for Platinum-Based Chemotherapy-Ineligible Elderly Patients with Locally Advanced HNSCC | no results available |
| Han 2022 | Treatment Outcomes for Radiation Therapy with Concurrent Carboplatin and Paclitaxel for Locally Advanced Head and Neck Cancer | abstract only |
| Mohamad 2022 | Intensified Radiation Therapy for Laryngeal Cancer: Oncologic and Functional Outcomes | lack of data for the cisplatin-unfit patients |
| Cirauqui Cirauqui 2022 | Real-world evidence of first-line cetuximab (CX) plus paclitaxel (PX) in recurrent or metastatic squamous cell carcinoma of the head and neck (SCCHN) | recurrent HNSCC; lack of population of interest |
| Patil 2022 | Long term toxicity and tolerance of concurrent docetaxel with radiotherapy in cisplatin-ineligible head and neck cancer patients | population overlap; another publication from the same trial already assessed |
| Kolkur 2022 | Long-term toxicity and tolerance of concurrent docetaxel with radiotherapy in cisplatin ineligible head and neck cancer patients | abstract only |
| Zereshkian 2022 | Nivolumab in squamous cell carcinomas of the head and neck (SCCHN): A real-world outcome study in Ontario, Canada | recurrent HNSCC; lack of population of interest |
| Locke 2022 | Efficacy and tolerability of a low dose weekly paclitaxel, carboplatin, cetuximab (PCC) regimen for metastatic or unresectable head and neck squamous cell carcinomas | |
| Tourneau 2022 | NANORAY-312: A phase III pivotal study of NBTXR3 activated by investigator's choice of radiotherapy alone or radiotherapy in combination with cetuximab for platinum-based chemotherapy-ineligible elderly patients with locally advanced head and neck squamous cell carcinoma | no published reults |
| NCT04892173 | NBTXR3 With or Without Cetuximab in LA-HNSCC | active study, no results available |
| Le Tourneau 2021 | Phase I Study of Novel Radioenhancer NBTXR3 Activated by Radiotherapy in Cisplatin-Ineligible Locally Advanced HNSCC Patients | active study, no results available |
| Tourneau 2021 | NBTXR3 activated by radiotherapy in cisplatin-ineligible locally advanced HNSCC patients | active study, no results available |
| Tourneau 2021 | Radiation enhancing hafnium oxide nanoparticles (NBTXR3) for the treatment of cisplatin-ineligible locally advanced HNSCC patients: a phase I dose expansion study | active study, no results available |
| Tourneau 2021 | Phase I study of functionalized hafnium oxide nanoparticles (NBTXR3) activated by radiotherapy in cisplatin-ineligible locally advanced HNSCC patients | active study, no results available |
| Le Tourneau 2020 | NBTXR3 Radiation Enhancing Hafnium Oxide Nanoparticles Activated By Radiotherapy In Cisplatin-Ineligible Patients With Locally Advanced HNSCC: A Phase I Trial | active study, no results available |
| Fryen 2022 | Moderately Hypofractionated Radiotherapy Without Chemotherapy in Elderly or Frail Patients With Head and Neck Cancer | lack of population of interest since age is not an absolute contraindication for cisplatin, this study was excluded because it may have included patients fit to received cisplatin-based regimens. |
| Abousaud 2022 | Institutional Comparison between Radiation with Concurrent Cisplatin Versus Carboplatin/paclitaxel for Squamous Cell Carcinoma of the Head and Neck (SCCHN) | abstract only |
| Carinato 2021 | Weekly Paclitaxel, Carboplatin, and Cetuximab as First-Line Treatment of Recurrent and/or Metastatic Head and Neck Squamous Cell Carcinoma for Patients Ineligible to Cisplatin-Based Chemotherapy: A Retrospective Monocentric Study in 60 Patients | recurrent HNSCC; lack of population of interest |
| Guigay 2021 | Avelumab for platinum-ineligible/refractory recurrent and/or metastatic squamous cell carcinoma of the head and neck: Phase Ib results from the JAVELIN Solid Tumor trial | recurrent HNSCC; lack of population of interest |
| Kainickal 2021 | Clinical profile and treatment outcomes of locally advanced carcinoma larynx treated with organ preservation approach: A retrospective analysis | lack of data for the cisplatin-unfit patients |
| Babbar 2021 | Carboplatin in combination with three weekly paclitaxel as first line therapy in patients with recurrent/metastatic head and neck cancers: A regional cancer center experience | recurrent HNSCC; lack of population of interest |
| Torneau 2023 | Novel Radioenhancer NBTXR3 Activated by Radiotherapy in Cisplatin-Ineligible Locally Advanced HNSCC Patients: Final Results of a Phase I Trial | abstract only |
| Gruenwald 2021 | Results of a randomized phase II study comparing pembrolizumab with methotrexate in elderly, frail or cisplatin-ineligible patients with relapsed or metastatic squamous cell carcinoma of the head and neck (RM-SCCHN) (ELDORANDO-AIO-KHT-0115) | unfinished study due to low recruitment rate |
| Mohan 2021 | High Dose Palliative-Intent Intensity Modulated Radiotherapy For Head and Neck Squamous Cell Carcinoma | lack of the population of interest |

| | | |
|---|---|---|
| Martinez Trufero 2019 | Final results of a phase II study of induction chemotherapy (CT) with paclitaxel (PTX) and panitumumab (P) followed by radiotherapy (RT) and P in patients (pts) with locally advanced head and neck cancer (LAHNC) no candidates to platinum: Study PANTERA | population overlap |
| Bernad 2017 | Activity of weekly paclitaxel−cetuximab chemotherapy in unselected patients with recurrent/metastatic head and neck squamous cell carcinoma: prognostic factors | recurrent HNSCC; lack of population of interest |
| Price 2021 | The effect of switching to carboplatin chemo-RT for cycle 2 in cisplatin-ineligible HNSCC patients | abstract only |
| Marinho 2021 | Safety and efficacy of concurrent carboplatin or cetuximab chemoradiation in cisplatin ineligible locally advanced head and neck squamous cell carcinoma | abstract only |
| Mitrofanov 2021 | Response rate after chemoembolization with followed radiation therapy in patients with locally advanced nasopharyngeal carcinoma | lack of data for the cisplatin-unfit patients |
| Sacco 2021 | Pembrolizumab plus cetuximab in patients with recurrent or metastatic head and neck squamous cell carcinoma: an open-label, multi-arm, non-randomised, multicentre, phase 2 trial | recurrent HNSCC; lack of population of interest |
| Chen 2021 | Combination of pembrolizumab and lenvatinib is a potential treatment option for heavily pretreated recurrent and metastatic head and neck cancer | lack of population of interest |
| Taguchi 2021 | Phase II trial of combination treatment with S-1/cetuximab in patients with platinum-ineligible recurrent and/or metastatic squamous cell carcinoma of the head and neck | recurrent HNSCC; lack of population of interest |
| Narvaez 2021 | Accelerated fractionation with concomitant boost vs. conventional radio-chemotherapy for definitive treatment of locally advanced squamous cell carcinoma of the head-and-neck (scchn) | lack of population of interest |
| Bauman 2021 | Randomized phase II trial of ficlatuzumab with or without cetuximab in pan-refractory, advanced head and neck squamous cell carcinoma (HNSCC) | recurrent HNSCC; lack of population of interest |
| Iglesias 2021 | Phase II multicenter randomized trial to assess the efficacy and safety of first line nivolumab in combination with paclitaxel in subjects with R/M HNSCC unable for cisplatin-based chemotherapy (NIVOTAX): A TTCC study | recurrent HNSCC; lack of population of interest |
| Klinghammer 2020 | ELDORANDO-Pembrolizumab compared with methotrexate in elderly, frail or cisplatin-ineligible patients with relapsed or metastatic squamous cell carcinoma of the head and neck (RM-SCCHN) (ELDORANDO-AIO-KHT-0115 ) | metastatic HNSCC; lack of population of interest |
| Bourhis 2021 | LBA35 Avelumab-cetuximab-radiotherapy versus standards of care in patients with locally advanced squamous cell carcinoma of head and neck (LA-SCCHN): Randomized phase III GORTEC-REACH trial | lack of population of interest |
| Tao 2018 | Avelumab-cetuximab-radiotherapy (RT) versus standards of care (SoC) in locally advanced squamous cell carcinoma of the head and neck (SCCHN): Safety phase of the randomized trial GORTEC 2017-01 (REACH) | population overlap, only safety outcomes |
| Tao 2019 | Avelumab-cetuximab-radiotherapy versus standards of care (SoC) in patients (pts) with locally advanced squamous cell carcinoma of head and neck (LA-SCCHN): Safety phase of randomized trial GORTEC 2017-01 (REACH) | population overlap, only safety outcomes |
| Tao 2020 | Avelumab–cetuximab–radiotherapy versus standards of care in locally advanced squamous-cell carcinoma of the head and neck: The safety phase of a randomised phase III trial GORTEC 2017-01 (REACH) | lack of outcomes of interest |
| Iganej 2020 | Radiotherapy With Weekly Cetuximab Versus Triweekly Carboplatin In The Definitive Treatment Of Cisplatin-Ineligible Patients With HPV-Associated Oropharyngeal Squamous Cell Carcinoma: One Institution's Retrospective Experience | abstract only |
| Menon 2020 | Clinical outcomes in patients with locally advanced head and neck cancer (LA HNSCC) receiving Cisplatin versus non-Cisplatin chemotherapy | lack of population of interest; all patients treated with cisplatin (high versus low dose) |
| Carinato 2020 | Weekly paclitaxel, carboplatin and cetuximab (PCC) as first-line treatment of recurrent and/or metastatic head & neck squamous cell carcinoma (R/M-HNSCC) for patients ineligible to cisplatin based chemotherapy | recurrent HNSCC; lack of population of interest |
| Forsyth 2020 | POPPY: A phase II trial to assess the efficacy and safety profile of pembrolizumab in patients with performance status 2 with recurrent or metastatic squamous cell carcinoma of the head and neck | active study, no results available |
| Weiss 2018 | Preliminary toxicity data from the combination of pembrolizumab and definitive-dose radiotherapy for locally advanced head and neck cancer with contraindication to cisplatin therapy | population overlap, study by Weiss 2020 already included |
| Ham 2020 | Methotrexate plus or minus cetuximab as first-line treatment in a recurrent or metastatic (R/M) squamous cell carcinoma population of the head and neck (SCCHN), unfit for cisplatin combination treatment, a phase Ib-randomized phase II study Commence | recurrent HNSCC; lack of population of interest |

| | | |
|---|---|---|
| Weiss 2020 | Progression-free survival, overall survival and immunophenotyping outcomes for patients with stage III-IV head and neck cancer and cisplatin contraindication treated with definitive radiotherapy plus pembrolizumab | population overlap, study by Weiss 2020 already included |
| Oppelt 2020 | nab-paclitaxel Monotherapy followed by Cetuximab and Radiation in Cisplatin-Unsuitable Patients with Locally Advanced Head and Neck Cancer: A Single-Arm, Multicenter Phase 2 Trial | abstract only, full publication already included |
| Sacco 2020 | An open-label, non-randomized, multi-arm, phase II trial evaluating pembrolizumab combined with cetuximab in patients (pts) with recurrent/metastatic (R/M) head and neck squamous cell carcinoma (HNSCC): updated results of cohort 1 analysis | abstract only, full publication of the same study already included |
| Sacco 2019 | An open label, nonrandomized, multi-arm, phase II trial evaluating pembrolizumab combined with cetuximab in patients with recurrent/metastatic (R/M) head and neck squamous cell carcinoma (HNSCC): Results of cohort 1 interim analysis | abstract only, full publication of the same study already included |
| Thanikachalam 2020 | Carboplatin versus cetuximab chemoradiation in cisplatin ineligible locally advanced head and neck squamous cell carcinoma | abstract only |
| Fayette 2019 | 90TiP FRAIL-IMMUNE: A multicenter, prospective, single arm phase II of the combination of durvalumab with carboplatin and paclitaxel as first-line treatment in patients with recurrent/metastatic squamous cell carcinoma of the head and neck not eligible to standard chemotherapy (GORTEC 2018-03) | recurrent HNSCC; lack of population of interest |
| Fayette 2010 | Paclitaxel is effective in relapsed head and neck squamous cell carcinoma: A retrospective study of 66 patients at a single institution | recurrent HNSCC; lack of population of interest |
| Sheth 2019 | Pembrolizumab and radiotherapy for locally advanced head and neck squamous cell carcinoma (LA-HNSCC) with contraindication to cisplatin therapy | abstract only |
| Hori 2019 | Real-world outcomes and prognostic factors in patients receiving nivolumab therapy for recurrent or metastatic head and neck carcinoma | lack of population of interest |
| Fung 2019 | A Real World Comparison of Cisplatin Versus Cetuximab Used Concurrently with Radiation in the Treatment of Locally Advanced Oropharyngeal Carcinoma | population overlap, study by Fung 2020 already included |
| Hamauchi 2015 | Safety and efficacy of concurrent carboplatin plus radiotherapy for locally advanced head and neck cancer patients ineligible for treatment with cisplatin | population overlap, study by Haumachi 2019 already included |
| Al-Saleh 2019 | Phase II/III Randomized Controlled Trial of Concomitant Hyperfractionated Radiotherapy plus Cetuximab (Anti-EGFR Antibody) or Chemotherapy in Locally Advanced Head and Neck Cancer | study does not specify if patients are cisplatin-unfit |
| Iwaki 2019 | Carboplatin plus paclitaxel in patients with recurrent/metastatic head and neck carcinoma | recurrent HNSCC; lack of population of interest |
| Maring 2017 | Carboplatin/paclitaxel-based radiochemotherapy is not inferior to cisplatin-based regimens in squamous cell carcinoma of head and neck (SCCHN) | recurrent HNSCC; lack of population of interest |
| Marín 2018 | Paclitaxel in combination with anti-EGFR therapy as induction chemotherapy for patients unfit for cisplatin with locally advanced head and neck squamous cell carcinoma (LA-HNSCC) | abstract only |
| Simha 2018 | Toxicity and tolerance of concurrent docetaxel with radiotherapy in cisplatin ineligible head and neck cancer patients | population overlap |
| Pêtre 2018 | Carboplatin in combination with weekly Paclitaxel as first-line therapy in patients with recurrent/metastatic head and neck squamous cell carcinoma unfit to EXTREME schedule | recurrent HNSCC; lack of population of interest |
| Barney 2018 | Carboplatin-based chemoradiation improves outcomes compared to cetuximab bioradiation in cisplatin ineligible locally advanced p16 negative head and neck cancer | abstract only |
| Barney 2018 | Clinical outcomes and prognostic factors in cisplatin versus cetuximab chemoradiation for locally advanced p16 positive oropharyngeal carcinoma | population overlap; another publication from the same center already assessed |
| Nawale 2017 | Toxicity, tolerance and outcomes of carboplatin based CTRT (concurrent chemoradiation) in head and neck cancers in patients who were not fit for cisplatin based CTRT | abstract only |
| Noronha 2017 | Carboplatin-based concurrent chemoradiation therapy in locally advanced head and neck cancer patients who are unfit for cisplatin therapy | population overlap; another publication from the same center already assessed |
| Talapatra 2016 | Nimotuzumab and intensity modulated radiation therapy (IMRT) in the concurrent setting of locally advanced head and neck cancers: Early results of a prospective trial in India | abstract only |
| Kumar 2015 | A phase 2 randomized study to compare short course palliative radiotherapy with short course concurrent palliative chemotherapy plus radiotherapy in advanced and unresectable head and neck cancer | lack of data for the cisplatin-unfit patients |

| | | |
|---|---|---|
| Delaruelle 2014 | Modified DCF (mDCF, docetaxel cisplatin 5FU) for patients with locally advanced squamous cell carcinoma of the head and neck (LASCCHN) unfit to DCF: A retrospective study | study does not specify if patients are cisplatin-unfit |
| Nguyen 2014 | 0-7-21 hypofractionated palliative radiation therapy in advanced head-and-neck cancers: A retrospective study | study does not specify if patients are cisplatin-unfit |
| Nguyen 2012 | Retrospective study of hypofractionated palliative radiation therapy for advanced head-and-neck cancers 0-7-21 | study does not specify if patients are cisplatin-unfit |
| Saigal 2014 | Concurrent radiotherapy with carboplatin and cetuximab for the treatment of medically compromised patients with locoregionally advanced head and neck squamous cell carcinoma | patients were treated with lose dose cisplatin; lack of population of interest |
| Saada 2012 | Docetaxel, carboplatin and fluoro-uracil (TCF) induction therapy in locally advanced head and neck squamous cell carcinoma (HNSCC) patients with contraindication for cisplatin based combination (TPF) | abstract only |
| Saad 2019 | Hypofractionated dose escalated VMAT; comparable efficacy and less toxicity than standard CCRT in HNC | abstract only |
| Saad 2020 | Comparison between hypo-fractionated dose-escalated volumetric modulated arc therapy and conventional concurrent chemo-radiation in locally advanced head and neck cancer: A pilot study | study does not specify if patients are cisplatin-unfit |
| Rivas 2012 | Retrospective analysis of the use of cetuximab (CTX) and paclitaxel (PTX) combination in recurrent and/or metastatic squamous cell carcinoma of the head and neck (R/M SCCHN) | study does not specify if patients are cisplatin-unfit |
| Hitt 2011 | Phase II study of the combination of cetuximab and weekly paclitaxel in the first-line treatment of patients with recurrent and/or metastatic squamous cell carcinoma of head and neck | recurrent HNSCC; lack of population of interest |
| Alongi 2012 | Prospective phase II trial of cetuximab plus VMAT-SIB in locally advanced head and neck squamous cell carcinoma: Feasibility and tolerability in elderly and chemotherapy-ineligible patients | lack of outcomes of interest |
| Sarkodie 2010 | Experience with nimorazole combined with radiotherapy for locally advanced squamous cell carcinoma of the head & neck (HNSCC) in a routine clinical practice | abstract only |
| Diaz De Corcuera 2009 | Clinical experience with cetuximab and paclitaxel combination in metastatic/recurrent squamous cell cancer carcinoma of the head and neck (SCCHN) - Retrospective analysis of a single institution | abstract only |
| Diaz De Corcuera 2009 | Weekly cetuximab and paclitaxel combination in metastatic/recurrent squamous cell cancer carcinoma of the head and neck (SCCHN): Clinical experience of a single institution | abstract only |
| Allan 2009 | Single institution review of radiotherapy plus cetuximab in head and neck cancer | abstract only |
| Porceddu 2004 | Postoperative chemoradiotherapy for high-risk head-and-neck squamous cell carcinoma | lack of data for the cisplatin-unfit patients |
| NCT00003592 | Phase III Randomized Trial of Methotrexate vs. Paclitaxel in Cisplatin-Ineligible Patients With Advanced Squamous Cell Carcinoma of the Head and Neck | study does not specify if patients are cisplatin-unfit |
| EUCTR2016-001331-12-DE | A randomized phase II study comparing pembrolizumab with methotrexate in elderly, frail or cisplatin-ineligible patients with head and neck cancers | population overlap, another publication from the same trial already assessed |
| NCT04282109 | Study to Assess the Efficacy and Safety of Nivolumab in Combination With Paclitaxel in Subjects With Head and Neck Cancer Unable for Cisplatin-based Chemotherapy (NIVOTAX) | active study, no results available |
| NCT05386550 | Phase III Xevinapant (Debio 1143) and Radiotherapy in Resected LA SCCHN, High Risk, Cisplatin-ineligible Participants (XRAY VISION) | active study, no results available |
| CTRI/2017/05/008700 | Docetaxel chemotherapy with radiation in head and neck cance | population overlap, another publication from the same trial already included |
| NCT00652613 | Study of 3-Dimensional Conformal Radiotherapy (3D-CRT) Versus Intensity Modulated Radiotherapy (IMRT) for Head and Neck Squamous Cell Carcinoma (HNSCC) | lack of population of interest |
| Motai 2021 | Clinical impact of weekly paclitaxel plus cetuximab is comparable to the EXTREME regimen for recurrent/metastatic head and neck squamous cell carcinoma | study does not specify if patients are cisplatin-unfit |
| NCT01216020 | Cetuximab Plus Radiotherapy Versus Cisplatin Plus Radiotherapy in Locally Advanced Head and Neck Cancer | unfinished study due to low recruitment rate |
| NCT03193931 | Study comparing pembrolizumab with methotrexate in elderly, frail or cisplatin-ineligible patients with head and neck cancers (ELDORANDO) | unfinished study due to low recruitment rate |

| | | |
|---|---|---|
| EUCTR2017-002676-87-PL | A Phase 3 Study of Nivolumab or Nivolumab plus Cisplatin, in Combination with Radiotherapy in Patients with Advanced Cancer of the Head and Neck | lack of outcomes of interest; only safety outcomes |
| NCT03349710 | Nivolumab or Nivolumab Plus Cisplatin, in Combination With Radiotherapy in Patients With Cisplatin-ineligible or Eligible Locally Advanced Squamous Cell Head and Neck Cancer | Results not available |
| Aguin 2023 | Real-world Data of Paclitaxel and Cetuximab in Recurrent/Metastatic Squamous Cell Carcinoma of the Head and Neck. | recurrent HNSCC; lack of population of interest |
| Zwicker 2011 | IMRT reirradiation with concurrent cetuximab immunotherapy in recurrent head and neck cancer. | lack of population of interest |
| Essa 2010 | Concurrent chemoradiation in locally advanced head and neck cancers: a comparative study of weekly Paclitaxel versus Cisplatin-based regimen | only abstract |
| Kim 2020 | Clinical outcomes of immune checkpoint inhibitors for patients with recurrent or metastatic head and neck cancer: real-world data in Korea. | study does not specify if patients are cisplatin-unfit |
| Rodríguez 2010 | Nimotuzumab plus radiotherapy for unresectable squamous-cell carcinoma of the head and neck. | lack of data for the cisplatin-unfit patients |
| Saba 2023 | Pembrolizumab and cabozantinib in recurrent metastatic head and neck squamous cell carcinoma: a phase 2 trial. | lack of data for the cisplatin-unfit patients |
| Schroeder 1998 | High response rates following paclitaxel/5-FU and simultaneous radiotherapy in advanced head and neck carcinoma | lack of data for the cisplatin-unfit patients |
| Lövey 2003 | Radiotherapy and concurrent low-dose paclitaxel in locally advanced head and neck cancer | lack of data for the cisplatin-unfit patients |
| Planting 2005 | Phase I study of concomitant chemoradiation with raltitrexed in locally advanced head and neck cancer | lack of data for the cisplatin-unfit patients |
| Vees 2006 | Carbogen Breathing Combined with Radical Radiotherapy in Advanced Head and Neck Cancer Patients with Severe Co-morbidities | study does not specify if patients are cisplatin-unfit |
| Carmona 2011 | Evaluation of toxicity with concurrent cetuximab and radiotherapy in head and neck cancer | abstract only |
| O'Sullivan 2011 | Excellent identical outcomes for radiation alone vs chemoradiation in minimal smoking HPV(+) N0-N2C oropharynx cancer patients | study does not specify if patients are cisplatin-unfit |
| Argiris 2011 | Phase II trial of cetuximab (C) and bevacizumab (B) in recurrent or metastatic squamous cell carcinoma of the head and neck (SCCHN): Final results | study does not specify if patients are cisplatin-unfit |
| Chew 2011 | Toxicity in combined modality treatment of HNSCC: Cisplatin versus cetuximab | abstract only |
| Bavan 2011 | Interim report of a phase II study of lapatinib and concurrent radiotherapy in patients with locally advanced head and neck cancer | lack of data for the cisplatin-unfit patients |
| Stevens 2011 | Retrospective study of palliative radiotherapy in newly diagnosed head and neck carcinoma | study does not specify if patients are cisplatin-unfit |
| Wilkins 2013 | Equivalence of cisplatin and carboplatin-based chemoradiation for locally advanced squamous cell carcinoma of the head and neck: A matched-pair analysis | Most patients were treated with induction chemotherapy regimens |
| Velasco Costa 2014 | Effectiveness and toxicity of cetuximab in head and neck cancer | abstract only |
| Yu 2014 | Patterns of care and treatment outcomes in elderly head and neck cancer patients treated with IMRT | lack of data for the cisplatin-unfit patients |
| Cvek 2014 | Hyperfractionated accelerated radiation therapy 70-75Gy in 5 weeks for advanced head and neck cancer: Single-institution experiences | study does not specify if patients are cisplatin-unfit |
| Cvek 2017 | Intensity modulated hyperfractionated accelerated radiotherapy to treat advanced head and neck cancer - Predictive factors of overall survival | lack of data for the cisplatin-unfit patients |
| Harrop 2015 | Chemoradiotherapy for poor/intermediate risk oropharyngeal carcinoma: First results of the ArCHIMEDEs study | lack of data for the cisplatin-unfit patients |
| Bonomo 2015 | Impact of intensity-modulated radiotherapy in the treatment of nasopharyngeal cancer: Single center experience | lack of data for the cisplatin-unfit patients |
| Skladowski 2015 | Is accelerated radiotherapy alone equivalent to chemoradiation in patients with moderate advanced HNSCC | lack of data for the cisplatin-unfit patients |
| Liu 2015 | High-risk HPV-related oropharyngeal cancer treated with adjuvant radiation therapy alone following surgical resection | Study does not specify if patients were cisplatin ineligible |

| | | |
|---|---|---|
| Bernard 2016 | Retrospective study of weekly paclitaxel-cetuximab (WPC) in unselected patients (p) with recurrent/metastatic head and neck squamous cell carcinoma (RM-SCCHN) | recurrent HNSCC; lack of population of interest |
| Fortin 2016 | Palliative radiation therapy for advanced head and neck carcinomas: A phase 2 study | study does not specify if patients are cisplatin-unfit |
| Spartacus 2016 | Re-irradiation in head and neck cancers: A single institutional experience | study does not specify if patients are cisplatin-unfit |
| Rastogi 2017 | Prospective evaluation of Intensity Modulated Radiation Therapy with Simultaneous Integrated Boost (IMRT-SIB) in head and neck squamous cell carcinoma in patients not suitable for chemo-radiotherapy | study does not specify if patients are cisplatin-unfit |
| Subramaniam 2017 | Radical radiotherapy for carcinoma of the larynx in the elderly: Functional and oncological outcomes from a tertiary cancer care center in India | study does not specify if patients are cisplatin-unfit |
| Harrington 2017 | Early safety from phase 1b/3, multicenter, open-label, randomized trial of talimogene laherparepvec (T-VEC) 1 pembrolizumab (pembro) for recurrent or metastatic squamous cell carcinoma of the head and neck (R/M SCCHN): MASTERKEY-232 | study does not specify if patients are cisplatin-unfit |
| Srinivas 2020 | Nimotuzumab with Intensity modulated radiation therapy (IMRT) in unresectable and platinum ineligible locally advanced head & neck cancer patients | population overlap; another publication from the same center already assessed |
| Aggarwal 2018 | A phase 1b/2a, multi-center, open-label study to evaluate the safety and efficacy of combination treatment with MEDI0457 (INO-3112) and durvalumab (MEDI4736) in patients with recurrent/metastatic human papilloma virus-associated head and neck squamous cell cancer | lack of data for the cisplatin-unfit patients |
| Orte 2018 | Suboptimal regimens in sequential treatment (ST) with ICT (induction chemotherapy) followed by CCRT (concomitant chemotherapy) in “real life” patients with locally advanced pharyngo-laryngeal squamous-cell carcinoma (LAPLSCC) and prognosis | lack of data for the cisplatin-unfit patients |
| Fanetti 2019 | Do comorbidities affect survival in head and neck cancer treated with Cetuximab and Radiotherapy? | study does not specify if patients are cisplatin-unfit |
| Hawkins 2018 | daptive chemoradiation therapy for head and neck cancer based on multiparametric MRI: Interim results of a prospective randomized trial | lack of data for the cisplatin-unfit patients |
| Martinez Trufero 2021 | Sequential chemotherapy regimen of induction with panitumumab and paclitaxel followed by radiotherapy and panitumumab in patients with locally advanced head and neck cancer unfit for platinum derivatives. The phase II, PANTERA/TTCC-2010-06 study | Induction chemotherapy |
| Barca 2023 | Electrochemotherapy as an Effective Alternative in the Treatment of Local Advanced Oral Squamous Cell Carcinoma: A Retrospective Analysis of Treated Cases | lack of data for the cisplatin-unfit patients |
| Cabezas-Camarero 2023 | Safety and preliminary activity of pembrolizumab-carboplatin-paclitaxel in heavily pretreated and/or fragile patients with PDL1-positive recurrent/metastatic head and neck cancer | recurrent HNSCC; lack of population of interest |
| Gogate 2022 | HSD73 First- and Second-Line Treatment Patterns in Recurrent or Metastatic Squamous Cell Carcinoma of the Head and Neck: A Chart Review Study in Three Countries | recurrent HNSCC; lack of population of interest |
| Martin-Jimenez 2022 | Paclitaxel Plus Cetuximab as Induction Chemotherapy for Patients With Locoregionally Advanced Head and Neck Squamous Cell Carcinoma Unfit for Cisplatin-Based Chemotherapy | Induction chemotherapy |
| Pathak 2022 | A retrospective analysis of patients administered neoadjuvant chemotherapy (NACT) with paclitaxel plus carboplatin with oral metronomic chemotherapy (OMCT) in locally advanced borderline resectable/technically unresectable s. | lack of data for the cisplatin-unfit patients |
| Patil 2022 | RMAC study: A randomized study for evaluation of metronomic adjuvant chemotherapy in recurrent head and neck cancers post salvage surgical resection in those who are ineligible for re-irradiation | lack of data for the cisplatin-unfit patients |
| Menon 2021 | 865MO RMAC study: A randomized study evaluating the efficacy of metronomic adjuvant chemotherapy in patients with recurrent head and neck cancers post salvage surgery, not eligible for re-irradiation | lack of data for the cisplatin-unfit patients |
| Blanchard 2022 | EVOLVE: Evaluating the Safety of De-escalated Head and Neck Irradiation in HPV Positive Oropharynx Cancer in Non/Minimal Smokers. | lack of data for the cisplatin-unfit patients |
| Ferris 2022 | Phase II Randomized Trial of Transoral Surgery and Low-Dose Intensity Modulated Radiation Therapy in Resectable p161 Locally Advanced Oropharynx Cancer: An ECOG-ACRIN Cancer Research Group Trial (E3311) | lack of data for the cisplatin-unfit patients |
| Ferris 2021 | Updated report of a phase II randomized trial of transoral surgical resection followed by lowdose or standard postoperative therapy in resectable p16+ locally advanced oropharynx cancer: A trial of the ECOG-ACRIN cancer research group (E3311) | lack of data for the cisplatin-unfit patients |
| Shenoy 2022 | Oral metronomic chemotherapy as a feasible preoperative therapy in advanced resectable oral cavity squamous cell carcinomas—a preliminary experience | lack of data for the cisplatin-unfit patients |

| | | |
|---|---|---|
| Shirasu 2020 | Efficacy and feasibility of induction chemotherapy with paclitaxel, carboplatin and cetuximab for locally advanced unresectable head and neck cancer patients ineligible for combination treatment with docetaxel, cisplatin, and 5-fluorouracil | study does not specify if patients are cisplatin-unfit, this study analyzes patients unfit for a regimen that combines three treatments, one of them being cisplatin. However, it is unclear if all included patients were ineligible exclusively for cisplatin |
| Yang 2021 | Stereotactic Body Radiation Therapy (SBRT) With or Without Daily Systemic Therapy for Re-Irradiation of Unresectable, Recurrent, Head and Neck Squamous Cell Carcinoma | lack of data for the cisplatin-unfit patients |
| Domb 2021 | Updated outcomes of split course radiotherapy in elderly or infirm patients with advanced cancers of the head and neck | lack of data for the cisplatin-unfit patients |
| Madabhavi 2021 | P-77 Immune Check Point Inhibitors in Head and Neck Cancers – An experience and literature review from a rural cancer centre of India | lack of data for the cisplatin-unfit patients |
| Hadi 2021 | Treatment outcome for local recurrent nasopharyngeal carcinoma in University Malaya Medical Centre from 2010-2017 | lack of data for the cisplatin-unfit patients |
| Perri 2021 | Electrochemotherapy as a first line treatment in recurrent squamous cell carcinoma of the oral cavity and oropharynx pdl-1 negative and/or with evident contraindication to immunotherapy: A randomized multicenter controlled trial | study does not specify if patients are cisplatin-unfit |
| Jiang 2021 | MSOR02 Presentation Time: 10:05 AM: Preliminary Results of 3D Printing Individual Template Assisted CT-Guided Radioactive I-125 Seed Interstitial Brachytherapy as a Salvage Therapy for Recurrent Head and Neck Squamous Carcinoma after External Beam Radiotherapy | study does not specify if patients are cisplatin-unfit |
| Culié 2021 | Upfront surgery or definitive radiotherapy for patients with p16-negative oropharyngeal squamous cell carcinoma. A GETTEC multicentric study | study does not specify if patients are cisplatin-unfit |
| Gogineni 2021 | Impact of stereotactic body radiation therapy on geriatric assessment and management for older patients with head and neck cancer using G8 | lack of population and outcomes of interest |
| Leddon 2021 | Adjuvant nivolumab following salvage resection in head and neck squamous cell carcinoma patients previously treated with definitive therapy: A single-arm phase II multi-institutional study | lack of data for the cisplatin-unfit patients |
| Oppelt 2021 | *nab*-Paclitaxel and cisplatin followed by cisplatin and radiation (Arm 1) and *nab*-paclitaxel followed by cetuximab and radiation (Arm 2) for locally advanced head and neck squamous-cell carcinoma: a multicenter, non-randomized phase 2 trial | Induction chemotherapy |
| Sambasivan 2020 | Outcomes of patients with locally advanced oral cavity SCC treated radically at Guys' Cancer Centre | lack of data for the cisplatin-unfit patients |
| Gogineni 2020 | Stereotactic body radiotherapy as primary treatment for elderly and medically inoperable patients with head and neck cancer | study does not specify if patients are cisplatin-unfit |
| Gogineni 2020 | Stereotactic Body Radiotherapy as Primary Treatment for Medically Unfit Patients with Head and Neck Cancer | study does not specify if patients are cisplatin-unfit |
| Aggarwal 2020 | Safety and efficacy of MEDI0457 plus durvalumab in patients (pts) with human papillomavirus-associated recurrent/metastatic head and neck squamous cell carcinoma (HPV+ R/M HNSCC) | lack of population of interest |
| Bonomo 2020 | Durvalumab with cetuximab and radiotherapy for locally advanced squamous cell carcinoma of the head and neck: A phase I/II trial | lack of population of interest |
| Iatì 2020 | Simultaneous Integrated Boost Radiotherapy in Unresectable Stage IV (M0) Head and Neck Squamous Cell Cancer Patients: Daily Clinical Practice | lack of population of interest |
| Fan 2020 | Last-line Local Treatment with the Quad Shot Regimen for Previously Irradiated Head and Neck Cancers | lack of population of interest |
| Schoenfeld 2020 | Neoadjuvant Nivolumab +/- Ipilimumab in Patients with Oral Cavity Cancer | lack of population of interest |
| Elbers 2020 | Immuno-radiotherapy with cetuximab and avelumab for advanced stage head and neck squamous cell carcinoma: Results from a phase-I trial | only safety outcomes; lack of outcomes of interest |
| EUCTR2008-000781-23-GB<br>Saloura 2014 | An Open-label, International, Multi-Center, Phase I/II, Dose-escalation Trial Investigating the Safety of Zalutumumab, a Human Monoclonal Epidermal Growth Factor Receptor Antibody in Combination with Radiotherapy, in Patients with Stage III, IVa or IVb Locally Advanced Squamous Cell Carcinoma of the Head and Neck Ineligible for Platinum based Chemotherapy - Zalutumumab in combination with radiotherapy in SCCHN patients ineligible for platinum based chemoth | results not available |
| Elbers 2018 | Radiotherapy with concurrent Avelumab and Cetuximab as primary treatment in patients with locally advanced squamous cell carcinoma of the head and neck: A phase-IB feasibility trial in patients unfit for cisplatin (NCT02938273) | population overlap; another publication from the same trial already assessed |

| | | |
|---|---|---|
| Dusi 2019 | Gefitinib along with methotrexate as palliative therapy in PS 3 and above in metastatic squamous cell carcinoma head and neck cancer patients | population overlap; another publication from the same trial already assessed |
| Wise-Draper 2019 | Interim analysis of a single arm phase II study of adjuvant nivolumab after salvage resection in head and neck squamous cell carcinoma patients previously treated with definitive therapy | population overlap; another publication from the same trial already assessed |
| Dickstein 2019 | Treatment Tolerability and Outcomes in Advanced-Age Oropharyngeal Cancer Patients | lack of population and outcomes of interest |
| Henk 2003 | Treatment of head and neck cancer with CHART and nimorazole: phase II study. | lack of population and outcomes of interest |
| Hua 2021 | Toripalimab plus intensity-modulated radiotherapy for recurrent nasopharyngeal carcinoma: an open-label single-arm, phase II trial. | lack of data for the cisplatin-unfit patients |
| UMIN000021870 | Clinical trial of concurrent chemoradiotherapy (CCRT) with S-1 in patients with advanced head and neck squamous cell carcinoma, who is not eligible for CCRT with Docetaxel, Cisplatin and 5-Fluorouracil (TPF) regimen | lack of population of interest |
| EUCTR2011-006017-34-FR | A clinical study with BYL719 in combination with cetuximab in patients with recurrent or metastatic head and neck squamous cell carcinoma | unfinished study |
| EUCTR2008-000781-23-GB | An Open-label, International, Multi-Center, Phase I/II, Dose-escalation Trial Investigating the Safety of Zalutumumab, a Human Monoclonal Epidermal Growth Factor Receptor Antibody in Combination with Radiotherapy, in Patients with Stage III, IVa or IVb Locally Advanced Squamous Cell Carcinoma of the Head and Neck Ineligible for Platinum based Chemotherapy - Zalutumumab in combination with radiotherapy in SCCHN patients ineligible for platinum based chemoth | unfinished study |
| EUCTR2004-002662-38-EE | A PHASE III RANDOMISED, STRATIFIED, PARALLEL-GROUP, MULTI-CENTRE, COMPARATIVE STUDY OF ZD1839 (IRESSA®) 250 MG AND 500 MG VERSUS METHOTREXATE FOR PREVIOUSLY TREATED PATIENTS WITH SQUAMOUS CELL CARCINOMA OF THE HEAD AND NECK - IMEX | study does not specify if patients are cisplatin-unfit |
| Grover 2016 | A single-institution comparison of cetuximab, carboplatin, and paclitaxel induction chemotherapy followed by chemoradiation (CRT) versus CRT for locally advanced squamous cell carcinoma of the head and neck (LA-SCCHN) | lack of population of interest |
| EUCTR2015-003011-38-AT | Pembrolizumab With or Without Talimogene Laherparepvec in Squamous Cell Carcinoma of the Head and Neck | study does not specify if patients are cisplatin-unfit |
| Taylor 1984 | A randomized comparison of high-dose infusion methotrexate versus standard-dose weekly therapy in head and neck squamous cancer | study does not specify if patients are cisplatin-unfit |
| Au 1998 | Activity of paclitaxel by three-hour infusion in Asian patients with metastatic undifferentiated nasopharyngeal cancer | lack of population of interest |
| Tao 2013 | Phase I trial of oral etoposide in combination with radiotherapy in head and neck squamous cell carcinoma - GORTEC 2004-02 | only safety outcomes; lack of outcomes of interest; another publication by Tao included |
| Holtzman 2015 | Surgery and radiation therapy or radiation therapy alone for salivary gland tumors: Outcomes update with up to 40 years of follow-up | lack of population of interest |
| EUCTR2013-003695-13-IT | non-blinded phase II study to compare the efficacy and safety of CetuGEX™ plus chemotherapy with cetuximab plus chemotherapy for the treatment of patients with stage III/IV recurrent and/or metastatic squamous cell carcinoma of the head and neck | lack of population of interest |
| EUCTR2009-016940-38-IT | Randomized, Double-blind, Multicenter Two-Stage Adaptive Phase 3 Study of Intravenous Administration of REOLYSIN (Reovirus Type 3 Dearing) in Combination with Paclitaxel and Carboplatin versus the Chemotherapy Alone in Patients with Metastatic or Recurrent Squamous Cell Carcinoma of the Head and Neck who have Progressed on or after Prior Platinum-Based Chemotherapy | includes patients who progressed on cisplatin; lack of population of interest |
| EUCTR2009-013402-14-IT | INduction chemoThERapy followed by CEtuximab Plus definiTive radiOtheRapy versus radiation plus cisplatin - INTERCEPTOR-TRIAL | results unavailable; low recruitment rate |
| EUCTR2008-005702-39-FR | Phase I/II study of Induction Chemotherapy with weekly RAD001, Carboplatine and Paclitaxel in Unresectable or Inoperable Locally Advanced Head and Neck Squamous Cell Carcinoma (HNSCC) - CAPRA | lack of population of interest |
| UMIN000021870 | Clinical trial of concurrent chemoradiotherapy (CCRT) with S-1 in patients with advanced head and neck squamous cell carcinoma, who is not eligible for CCRT with Docetaxel, Cisplatin and 5-Fluorouracil (TPF) regimen | lack of population of interest |
| De Mulder 1994 | A phase II study of pirarubicin in patients with advanced recurrent head and neck squamous cell carcinoma. | lack of population of interest |
| Mehanna 2019 | Radiotherapy plus cisplatin or cetuximab in low-risk human papillomavirus-positive oropharyngeal cancer (De-ESCALaTE HPV): an open-label randomised controlled phase 3 trial | lack of population of interest |
| Gillson 2019 | Radiotherapy plus cetuximab or cisplatin for human papillomavirus (HPV)-positive oropharyngeal cancer: a randomized, multicenter, non-inferiority clinical trial | lack of population of interest |

**Table S8: Cisplatin ineligibility criteria according to the information available in studies included in the systematic review.**

| Study | Main cisplatin ineligibility criteria (N) | | | |
|---|---|---|---|---|
| | **Comorbidities** | **Impaired renal function** | **Otological disorders** | **Socioeconomic status/older age/poor performance status/weight loss** |
| **Addeo 2019** | **Comorbidity**<br>-Active HCV (N=7)<br>-Recurrent pulmonary infections (N=5)<br>-Uncontrolled diabetes (N=11)<br>-Hypertension or unstable cardiac disease (N=12)<br>-Anemia >grade 2 (N=9) | **Renal dysfunction**<br>-CCR <50 mL/min (N=5)<br>-CCR >50 but <100 mL/min (N=9) | **Otological disorders**<br>-Medical history: pre-existant hearing loss or tinnitus >grade 2 (N=4)<br>-Abnormal audiometry within an audible frequency (N=5) | **Socioeconomic status**<br>-No social support (N=10)<br>-No support at home (N=6)<br>**BMI**<br>≥25 (overweight) (N=6)<br>≥18.5 but <25 (normal ) (N=25)<br><18.5 (underweight) (N=33)<br>**Weight loss and nutritional status**<br>Involuntary weight loss ≥20% (N= 16) |
| **Agarwal 2011** | **Major comorbidities** (N=9) | **Impaired renal function** (N=22) | - | **Older age** (N=8) |
| **Beckham 2019** | Not described | | | |
| **Corry 2017** | **-Comorbidities associated with ECOG OPS 2** (N=5)<br>**-Inability to tolerate intravenous hydration** (N=3) | **Renal impairment** (N=3) | **-Sensorineural hearing impairment** (N=34)<br>**-Sensorineural hearing impairment + renal impairment** (N=1)<br>**-Sensorineural hearing impairment + severe tinnitus** (N=6)<br>**-Severe tinnitus** (N=10) | - |
| **Fung 2020** | Not described | | | |
| **Hamauchi 2019** | **-Age>76 years** (N=17)<br>**-Cardiac dysfunction (history of unstable angina pectoris, myocardial infarction, or chronic heart failure** (N=10)<br>**-Performance status (PS) 2** (N=3) | **Renal impairment [creatinine clearance (CCr)<60 mL/min]** (N=21) | **Neurologic impairment (peripheral neuropathy or hearing impairment)** (N=5) | - |
| **Han 2023** | - | **Hearing, renal, or neuropathy** (N=30) | - | **-Frailty (extensive medical comorbidities and/or social issues)** (N=27)<br>**-Patient choice** (N=5) |
| **Imai 2022** | Not described | | | |
| **Magnes 2021** | Not described | | | |

| | | | | |
|---|---|---|---|---|
| **Maring 2018** | Not described | | | |
| **Nassif 2022** | Not described | | | |
| **Patil 2023** | **-Organ dysfunction of grade 2 or higher on the basis of the National Cancer Institute Common Toxicity Criteria (NCI CTC) version 4.0, such as hearing loss and tinnitus and neurologic disorders** (N=NA)<br>**- Hypersensitivity to cisplatin** (N=NA)<br>**-Borderline organ function or comorbidities precluding the use of cisplatin, for example,**<br>**reduction in left ventricular ejection fraction to below 50%, which could preclude cisplatin hydratio**n (N=NA)<br>**-Uncontrolled or poorly controlled hypertension** (N=NA) | **-Calculated creatinine clearance of , 50 mL/min** (N=NA)<br>**-Borderline organ function or comorbidities precluding the use of cisplatin, for example, reduction in left ventricular ejection fraction to below 50%, which could preclude cisplatin hydration** (N=NA)<br>**-Renal dysfunction (noncorrectable) with high creatinine even with creatinine clearance of <50 mL/min** (N=NA)<br>**- Concomitant use of nephrotoxic drugs required for a concurrent medical condition** (N=NA)<br>**-Calculated creatinine clearance of , 50 mL/min** (N=NA)<br>**-Borderline organ function or comorbidities precluding the use of cisplatin, for example, reduction in left ventricular ejection fraction to below 50%, which could preclude cisplatin hydration** (N=NA)<br>**-Renal dysfunction (noncorrectable) with high creatinine even with creatinine clearance of <50 mL/min** (N=NA)<br>**- Concomitant use of nephrotoxic drugs required for a concurrent medical condition** (N=NA) | | **-ECOG PS 2** (N=NA)<br>**-Loss of . 10% of baseline body weight in preceding 6 month**s (N=NA)<br>**-Malnourished status defined by the body mass index , 16 kg/m2** (N=NA) |
| **Pryor 2009** | **-Multiple comorbidities** (N=1)<br>**-Cardiac disease** (N=3) | **Renal impairment** (N=5) | **Hearing impairment** (N=4) | - |
| **Rades 2023** | - | **Impaired renal function** (N=NA) | - | - |
| **Rambeau 2017** | **-Cardiac past history** (N=25)<br>**-Poor general condition** (N=23)<br>**-Other active cancer** (N=8)<br>**-Vascular past history** (N=7)<br>**-Respiratory past history** (N=7)<br>**-Liver failure (cirrhosis)** (N=6)<br>**-Thrombocytopenia** (N=1) | **Renal failure** (N=8) | **-Neuropathy** (N=3)<br>**-Hypoacusia** (N=1) | **-Expected compliance difficulties** (N=9)<br>**-Previous chemotherapy with poor tolerance** (N=2)<br>**-Older age** (N=6) |
| **Saigal 2014** | - | **Chronic renal insufficiency** (N=NA) | **Hearing loss** (N=NA) | **-Older age and/or frailty** (N=NA)<br>**-Poor functional reserve** (N=NA)<br>**-Poor social support** (N=NA) |
| **Srinivas 2020** | Not described | | | |
| **Sun 2022** | Not described | | | |
| **Swiecicki 2020** | **Comorbidities that precluded treatment with standard platinum-based chemotherapy** (N=NA) | **Creatinine clearance < 30 cc/min** (N=NA) | - | **Older age** (N=NA) |

| | | | | |
|---|---|---|---|---|
| **Tao 2023** | **Cardiac function incompatible with hyperhydration with significant heart disease** (N=NA) | **Calculated creatinine clearance <60 ml/min** (N=NA) | **-Peripheral neuropathy grade 2** (N=NA)<br>**-Sensorineural hearing loss grade 2 (confirmed by audiogram)** (N=NA) | **-Age 75 years** (N=NA)<br>-Age 71-74 years and PS 1 and/or G8 score 14 (N=NA)<br>**-General clinical status incompatible with high-dose cisplatin and RT according to the investigator** (N=NA) |
| **Van Der Linden 2013** | - | **Impaired renal function** (N=8) | **-** | **Weakened condition** (N=8)<br>**Older age** (N=5) |
| **Ueki 2023** | - | **Less than 75 years old and had poor renal function (30–60 mL/min eGFR** (N=17)<br>**-75 years old or older with eGFR ≥ 40 mL/min** (N=13) | - | - |
| **Weiss 2020** | **Diabetes** (N=2) | **Renal function** (N=5) | **-Hearing impairmen**t (N=14)<br>**-Tinnitus** (N=6)<br>**-Neuropathy** (N=2) | s |
| **Ye 2013** | **-Cardiac dysfunction** (N=NA)<br>**-Inadequate marrow function (based on evaluation of blood counts, at the discretion of the treating medical oncologist)** (N=NA) | **-Inadequate renal function (creatinine clearance less than 45 ml/min)** (N=NA) | **-Neuropathy or hearing impairment** (N=NA) | **Poor performance status (PS) ECOG 3 or 4)** (N=NA) |

N: Number of patients; (-) criteria not assessed or no patients fit the specific criteria; ECOG: Eastern Cooperative Oncology Group; GFR: glomerular filtration rate.

**Table S9: Disease-free survival (DFS) at 1 year (A); and 2 years (B), according to the treatment regimen.**

**(A) 1-year DFS**

| Study | N | Total | Proportion | Treatment regimen |
|---|---|---|---|---|
| Swiecicki 2020 | 10 | 21 | 47.6% | CTX-RT |
| Ye 2013 | 58 | 87 | 66.7% | CTX-RT |

**(B) 2-year DFS**

| Study | N | Total | Proportion | Treatment regimen |
|---|---|---|---|---|
| Agarwal 2011 | 11 | 37 | 29.5% | CTX-RT |
| Swiecicki 2020 | 8 | 21 | 38.1% | CTX-RT |
| Patil 2023 | 53 | 176 | 30.3% | RT alone |
| Patil 2023 | 76 | 180 | 42.2% | DTX-RT |

N: number of patients who achieved certain response or rate; Total: total number of patients; CTX: cetuximab; RT: radiotherapy; Pembro: pembrolizumab; DTX: docetaxel.

**Table S10: Locoregional control rate (LRC) at 1 year/15 months (A); 2 years (B), 3 years (C); and 4 years (D), according to the treatment regimen.**

**(A) LRC at one year or 15-months**

| Study | N | Total | Proportion | Treatment regimen | Data cutoff |
|---|---|---|---|---|---|
| Tao 2023 | 33 | 56 | 58.9% | CTX-RT | 15 months LRC |
| Ye 2013 | 62 | 87 | 71.3% | CTX-RT | 1-year LRC |
| Tao 2023 | 34 | 57 | 59.6% | Pembro-RT | 15 months LRC |
| Rades 2023 | 35 | 45 | 78% | CB-RT | 1-year LRC |

**(B) 2-year LRC**

| Study | N | Total | Proportion | Treatment regimen |
|---|---|---|---|---|
| Agarwal 2011 | 13 | 37 | 35.5% | CTX-RT |
| Rades 2023 | 31 | 45 | 69% | CB-RT |

**(C) 3-year LRC**

| Study | N | Total | Proportion | Treatment regimen |
|---|---|---|---|---|
| Hamauchi 2019 | 10 | 18 | 58% | CTX-RT |
| Hamauchi 2019 | 16 | 29 | 56% | CB-RT |
| Rades 2023 | 29 | 45 | 65% | CB-RT |
| Saigal 2014 | 5 | 16 | 31.3% | CB-RT + CTX |
| Nassif 2022 | 27 | 30 | 88.3% | CB-RT + paclitaxel |

**(D) 4-year LRC**

| Study | N | Total | Proportion | Treatment regimen |
|---|---|---|---|---|
| Corry 2017 | 49 | 60 | 81.7% | CB-RT+CTX |

N: number of patients who achieved certain response or rate; Total: total number of patients; CTX: cetuximab; RT: radiotherapy; CB: carboplatin; Pembro: pembrolizumab.

**Table S11: Incidence of distant recurrence/metastases (A); and locoregional failure (B), according to the regimen used.**

**(A) Incidence of distant recurrence or metastases according to the regimen used.**

| Study | N | Total | Proportion | Treatment regimen |
|---|---|---|---|---|
| Addeo 2019 | 9 | 64 | 14% | CTX-RT |
| Beckham 2019 | 15 | 61 | 25% | CTX-RT |
| Hamauchi 2019 | 4 | 18 | 22.2% | CTX-RT |
| Ye 2013 | 16 | 87 | 18.4% | CTX-RT |
| Tao 2023 | 10 | 65 | 15.4% | CTX-RT |
| Imai 2022 | 38 | 88 | 43.2% | CTX-RT |
| Beckham 2019 | 9 | 74 | 12% | CB-RT |
| Hamauchi 2019 | 3 | 29 | 10.3% | CB-RT |
| Han 2023 | 6 | 65 | 9.4% | CB-RT + paclitaxel |
| Saigal 2014 | 3 | 16 | 18.8% | CB-RT + CTX |
| Tao 2023 | 9 | 66 | 13.6% | Pembro-RT |
| Patil 2023 | 31 | 180 | 17.2% | DTX-RT |
| Patil 2023 | 28 | 176 | 15.9% | RT alone |

**(B) Locoregional failure according to the regimen used.**

| Study | N | Total | Proportion | Treatment regimen |
|---|---|---|---|---|
| Corry 2017 | 8 | 60 | 13.3% | CTX-RT |
| Hamauchi 2019 | 4 | 18 | 22.2% | CTX-RT |
| Hamauchi 2019 | 7 | 29 | 24.1% | CB-RT |
| Han 2023 | 6 | 65 | 8.8% | CB-RT + paclitaxel |
| Maring 2018 | 3 | 23 | 13% | CB-RT + paclitaxel |
| Saigal 2014 | 5 | 16 | 31.3% | CB-RT + CTX |

N: number of patients; CTX: cetuximab; RT: radiotherapy; CB: carboplatin; Pembro: pembrolizumab; DTX: docetaxel.

**Table S12: Objective response rate (A); and disease control rate (B) according to the regimen used.**

**(A) Objective response rate (ORR) according to the regimen used.**

| Study | N | Total | Proportion | Treatment regimen |
|---|---|---|---|---|
| Addeo 2019 | 43 | 64 | 67.2% | CTX-RT |
| Agarwal 2011 | 29 | 30 | 96.7% | CTX-RT |
| Rambeau 2017 | 41 | 88 | 46.6% | CTX-RT |
| Pryor 2009 | 7 | 9 | 77.8% | CTX-RT |
| Saigal 2014 | 14 | 15 | 93.3% | CTX-CB-RT |
| Ueki 2023 | 27 | 30 | 90% | CB-RT |
| Srinivas 2020 | 18 | 21 | 85.7% | Nimo-RT |
| Patil 2023 | 64 | 176 | 36.4% | RT alone |
| Patil 2023 | 71 | 180 | 39.4% | DXT-RT |

**(B) Disease control rate (DCR) according to the regimen used.**

| Study | N | Total | Proportion | Treatment regimen |
|---|---|---|---|---|
| Addeo 2019 | 48 | 64 | 75% | CTX-RT |
| Agarwal 2011 | 30 | 30 | 100% | CTX-RT |
| Rambeau 2017 | 41 | 88 | 46.6% | CTX-RT |
| Srinivas 2020 | 19 | 21 | 90.5% | Nimo-RT |
| Patil 2023 | 64 | 176 | 36.4% | RT alone |
| Patil 2023 | 72 | 180 | 40% | DXT-RT |
| Ueki 2023 | 27 | 30 | 90% | CB-RT |

N: number of patients; CTX: cetuximab; RT: radiotherapy; CB: carboplatin; Pembro: pembrolizumab; DTX: docetaxel; Nimo: nimotuzumab.

**Table S13: Incidence of commonly reported adverse events among patients treated with carboplatin-RT (A); cetuximab-RT (B); docetaxel-RT (C); pembrolizumab-RT (D); nimotuzumab-RT (E); carboplatin-RT plus paclitaxel (F); and carboplatin-RT plus cetuximab (G).**

**(A) Carboplatin-RT**

| Study/AEs | Cases (N) | Total (N) | Proportion | AEs Grade |
|---|---|---|---|---|
| **Dry mouth** | | | | |
| Hamauchi 2019 | 28 | 29 | 97% | All grades |
| Hamauchi 2019 | 28 | 29 | 97% | Grade 1-2 |
| Hamauchi 2019 | 0 | 29 | 0 | Grade ≥3 |
| **RT dermatitis** | | | | |
| Hamauchi 2019 | 29 | 29 | 100% | All grades |
| Hamauchi 2019 | 27 | 29 | 93% | Grade 1-2 |
| Hamauchi 2019 | 2 | 29 | 7% | Grade ≥3 |
| **Mucositis** | | | | |
| Hamauchi 2019 | 29 | 29 | 100% | All grades |
| Hamauchi 2019 | 13 | 29 | 45% | Grade 1-2 |
| Hamauchi 2019 | 16 | 29 | 55% | Grade ≥3 |
| Ueki 2023 | 28 | 30 | 93.3% | All grades |
| Ueki 2023 | 15 | 30 | 50% | Grade 1-2 |
| Ueki 2023 | 14 | 30 | 46.7% | Grade ≥3 |

**(B) Cetuximab-RT**

| Study/AEs | Cases (N) | Total (N) | Proportion | AEs Grade |
|---|---|---|---|---|
| **Skin toxicity** | | | | |
| Addeo 2019 | 33 | 64 | 51.6% | All grades |
| Addeo 2019 | 25 | 64 | 39.1% | Grade 1-2 |
| Addeo 2019 | 8 | 64 | 12.5% | Grade ≥3 |
| Hamauchi 2019 | 18 | 18 | 100% | All grades |
| Hamauchi 2019 | 17 | 18 | 94.4% | Grade 1-2 |
| Hamauchi 2019 | 1 | 18 | 5.6% | Grade ≥3 |
| Imai 2022 | 3 | 88 | 3.4% | All grades |
| Swiecicki 2020 | 16 | 21 | 76.2% | All grades |
| Swiecicki 2020 | 12 | 21 | 57.1% | Grade 1-2 |
| Swiecicki 2020 | 4 | 21 | 19% | Grade ≥3 |
| Ye 2013 | 10 | 87 | 11.5% | Grade ≥3 |
| Pryor 2009 | 13 | 13 | 100% | All grades |
| Pryor 2009 | 7 | 13 | 53.8% | Grade 1-2 |
| Pryor 2009 | 6 | 13 | 46.2% | Grade ≥3 |
| Agarwal 2011 | 15 | 37 | 41% | Grade ≥3 |
| Tao 2023 | 41 | 65 | 63.1% | All grades |
| Tao 2023 | 31 | 65 | 47.7% | Grade 1-2 |
| Tao 2023 | 10 | 65 | 15.4% | Grade ≥3 |
| **RT dermatitis** | | | | |
| Addeo 2019 | 20 | 64 | 31.3% | All grades |
| Addeo 2019 | 13 | 64 | 20.3% | Grade 1-2 |
| Addeo 2019 | 7 | 64 | 10.9% | Grade ≥3 |
| Hamauchi 2019 | 18 | 18 | 100% | All grades |
| Hamauchi 2019 | 9 | 18 | 50% | Grade 1-2 |
| Hamauchi 2019 | 9 | 18 | 50% | Grade ≥3 |
| Ye 2013 | 18 | 87 | 20.7% | Grade ≥3 |
| Tao 2023 | 57 | 65 | 87.7% | All grades |
| Tao 2023 | 19 | 65 | 29.2% | Grade 1-2 |
| Tao 2023 | 38 | 65 | 58.4% | Grade ≥3 |
| **Dysphagia** | | | | |
| Addeo 2019 | 23 | 64 | 35.9% | All grades |
| Addeo 2019 | 16 | 64 | 25% | Grade 1-2 |
| Addeo 2019 | 7 | 64 | 10.9% | Grade ≥3 |
| Hamauchi 2019 | 14 | 18 | 77.8% | All grades |

| | | | | |
|---|---|---|---|---|
| Hamauchi 2019 | 8 | 18 | 44.4% | Grade 1-2 |
| Hamauchi 2019 | 6 | 18 | 33.3% | Grade ≥3 |
| Swiecicki 2021 | 14 | 21 | 66.7% | All grades |
| Swiecicki 2021 | 9 | 21 | 42.9% | Grade 1-2 |
| Swiecicki 2021 | 5 | 21 | 23.8% | Grade ≥3 |
| Pryor 2009 | 13 | 13 | 100% | All grades |
| Pryor 2009 | 6 | 13 | 46.2% | Grade 1-2 |
| Pryor 2009 | 7 | 13 | 53.8% | Grade ≥3 |
| **Mucositis** | | | | |
| Addeo 2019 | 14 | 64 | 21.9% | All grades |
| Addeo 2019 | 11 | 64 | 17.2% | Grade 1-2 |
| Addeo 2019 | 3 | 64 | 4.7% | Grade ≥3 |
| Hamauchi 2019 | 18 | 18 | 100% | All grades |
| Hamauchi 2019 | 4 | 18 | 22.2% | Grade 1-2 |
| Hamauchi 2019 | 14 | 18 | 77.8% | Grade ≥3 |
| Imai 2020 | 9 | 88 | 10.2% | All grades |
| Ye 2013 | 20 | 87 | 23% | Grade ≥3 |
| Swiecicki 2021 | 19 | 21 | 90.5% | All grades |
| Swiecicki 2021 | 11 | 21 | 52.4% | Grade 1-2 |
| Swiecicki 2021 | 8 | 21 | 38.1% | Grade ≥3 |
| Pryor 2009 | 13 | 13 | 100% | All grades |
| Pryor 2009 | 3 | 13 | 23.1% | Grade 1-2 |
| Pryor 2009 | 10 | 13 | 76.9% | Grade ≥3 |
| Rambeau 2017 | 87 | 88 | 98.9% | All grades |
| Rambeau 2017 | 46 | 88 | 52.3% | Grade 1-2 |
| Rambeau 2017 | 20 | 88 | 22.7% | Grade ≥3 |
| Tao 2023 | 58 | 65 | 89.2% | All grades |
| Tao 2023 | 20 | 65 | 30.8% | Grade 1-2 |
| Tao 2023 | 38 | 65 | 58.5% | Grade ≥3 |

**(C) Docetaxel-RT**

| Study | Cases (N) | Total (N) | Proportion | AEs Grade |
|---|---|---|---|---|
| **Skin toxicity** | | | | |
| Patil 2023 | 154 | 179 | 86% | All grades |
| Patil 2023 | 127 | 179 | 70.1% | Grade 1-2 |
| Patil 2023 | 27 | 179 | 15.1% | Grade ≥3 |
| **Mucositis** | | | | |
| Patil 2023 | 177 | 179 | 98.9% | All grades |
| Patil 2023 | 88 | 179 | 49.2% | Grade 1-2 |
| Patil 2023 | 89 | 179 | 49.7% | Grade ≥3 |
| **Dysphagia** | | | | |
| Patil 2023 | 161 | 179 | 89.9% | All grades |
| Patil 2023 | 72 | 179 | 40.2% | Grade 1-2 |
| Patil 2023 | 89 | 179 | 49.7% | Grade ≥3 |

**(D) Pembrolizumab-RT**

| Study | Cases (N) | Total (N) | Proportion | AEs Grade |
|---|---|---|---|---|
| **Anemia** | | | | |
| Tao 2023 | 4 | 66 | 6.1% | All grades |
| Tao 2023 | 3 | 66 | 4.5% | Grade 1-2 |
| Tao 2023 | 1 | 66 | 1.5% | Grade ≥3 |
| Weiss 2020 | 11 | 29 | 37.4% | All grades |
| Weiss 2020 | 11 | 29 | 37.9% | Grade 1-2 |
| Weiss 2020 | 0 | 29 | 0 | Grade ≥3 |
| **Mucositis** | | | | |
| Tao 2023 | 60 | 66 | 90.1% | All grades |
| Tao 2023 | 39 | 66 | 59.1% | Grade 1-2 |
| Tao 2023 | 21 | 66 | 31.2% | Grade ≥3 |
| Weiss 2020 | 24 | 29 | 82.8% | All grades |
| Weiss 2020 | 17 | 29 | 58.6% | Grade 1-2 |
| Weiss 2020 | 7 | 29 | 24.1% | Grade ≥3 |
| **Dysphagia** | | | | |
| Weiss 2020 | 12 | 29 | 41.4% | All grades |
| Weiss 2020 | 10 | 29 | 34.5% | Grade 1-2 |
| Weiss 2020 | 2 | 29 | 6.9% | Grade ≥ 3 |
| **Diarrhea** | | | | |
| Tao 2023 | 4 | 66 | 6.1% | All grades |
| Tao 2023 | 4 | 66 | 6.1% | Grade 1-2 |
| Tao 2023 | 0 | 66 | 0 | Grade ≥3 |

**(E) Nimotuzumab-RT**

| Study | Cases (N) | Total (N) | Proportion | AEs Grade |
|---|---|---|---|---|
| **Anemia** | | | | |
| Srinivas 2020 | 4 | 21 | 19.1% | All grades |
| Srinivas 2020 | 4 | 21 | 19.1% | Grade 1-2 |
| Srinivas 2020 | 0 | 21 | 0 | Grade ≥3 |
| **Mucositis** | | | | |
| Srinivas 2020 | 7 | 21 | 33.3% | All grades |
| Srinivas 2020 | 7 | 21 | 33.3% | Grade 1-2 |
| Srinivas 2020 | 0 | 21 | 0 | Grade ≥3 |
| **Dysphagia** | | | | |
| Srinivas 2020 | 4 | 21 | 19.1% | All grades |
| Srinivas 2020 | 4 | 21 | 19.1% | Grade 1-2 |
| Srinivas 2020 | 0 | 21 | 0 | Grade ≥ 3 |
| **Diarrhea** | | | | |
| Srinivas 2020 | 3 | 21 | 14.3% | All grades |
| Srinivas 2020 | 3 | 21 | 14.3% | Grade 1-2 |
| Srinivas 2020 | 0 | 21 | 0 | Grade ≥3 |

**(F) Carboplatin-RT plus paclitaxel**

| Study | Cases (N) | Total (N) | Proportion | AEs Grade |
|---|---|---|---|---|
| **Skin toxicity** | | | | |
| Maring 2018 | 21 | 23 | 91.3% | All grades |
| Maring 2018 | 14 | 23 | 60.9% | Grade 1-2 |
| Maring 2018 | 7 | 23 | 30.4% | Grade ≥3 |
| **Mucositis** | | | | |
| Maring 2018 | 21 | 23 | 91.3% | All grades |
| Maring 2018 | 18 | 23 | 78.3% | Grade 1-2 |
| Maring 2018 | 3 | 23 | 13% | Grade ≥3 |
| **Dysphagia** | | | | |
| Maring 2018 | 23 | 23 | 100% | All grades |
| Maring 2018 | 22 | 23 | 95.7% | Grade 1-2 |
| Maring 2018 | 1 | 23 | 4.3% | Grade ≥3 |

**(G) Carboplatin-RT plus cetuximab**

| Study | Cases (N) | Total (N) | Proportion | AEs Grade |
|---|---|---|---|---|
| **Dry mouth** | | | | |
| Corry 2017 | 48 | 58 | 82.8% | All grades |
| Corry 2017 | 42 | 58 | 72.4% | Grade 1-2 |
| Corry 2017 | 6 | 58 | 10.3% | Grade ≥3 |
| **Skin toxicity** | | | | |
| Corry 2017 | 45 | 58 | 77.6% | All grades |
| Corry 2017 | 36 | 58 | 62.1% | Grade 1-2 |
| Corry 2017 | 9 | 58 | 15.5% | Grade ≥3 |
| Saigal 2014 | 16 | 16 | 100% | All grades |
| Saigal 2014 | 16 | 16 | 100% | Grade 1-2 |
| Saigal 2014 | 0 | 16 | 0 | Grade ≥3 |
| **RT dermatitis** | | | | |
| Corry 2017 | 48 | 58 | 82.8% | All grades |
| Corry 2017 | 28 | 58 | 48.3% | Grade 1-2 |
| Corry 2017 | 20 | 58 | 34.5% | Grade ≥3 |
| Saigal 2014 | 16 | 16 | 100% | All grades |
| Saigal 2014 | 10 | 16 | 62.5% | Grade 1-2 |
| Saigal 2014 | 6 | 16 | 37.5% | Grade ≥3 |
| **Mucositis** | | | | |
| Saigal 2014 | 16 | 16 | 100% | All grades |
| Saigal 2014 | 9 | 16 | 56.3% | Grade 1-2 |
| Saigal 2014 | 7 | 16 | 43.4% | Grade ≥3 |
| Corry 2017 | 57 | 58 | 98.3% | All grades |
| Corry 2017 | 5 | 58 | 8.6% | Grade 1-2 |
| Corry 2017 | 52 | 58 | 89.7% | Grade ≥3 |

N: Number of patients; AEs: adverse events; RT: radiotherapy

**Table S14: Quality assessment for studies included in this systematic review using: risk of bias summary for non-randomized studies (ROBINS-I) tool for retrospective cohorts and prospective studies (A), and risk-of-bias 2 tool for randomized clinical trials (RoB 2) (B).**

**(A) Risk of bias summary for non-randomized studies (ROBINS-I).**

| Study | Bias due to confounding | Bias in selection of participants | Bias in classification of interventions | Bias due to deviations from intended interventions | Bias due to missing data | Bias in measurment of outcomes | Bias in selection of reported results | Overall risk of bias judgment |
|---|---|---|---|---|---|---|---|---|
| **Addeo 2019** | Serious[a] | Low | Low | Low | Low | Low | Moderate[b] | Serious |
| **Agarwal 2011** | Moderate | Low | Low | Low | Low | Low | Low[c] | Moderate |
| **Beckham 2019** | Moderate | Low | Low | Low | Low | Low | Low[c] | Moderate |
| **Corry 2017** | Moderate[c] | Low | Low | Low | Low | Low | Low[c] | Moderate |
| **Fung 2020** | Moderate | Low | Low | Low | Low | Low | Low[c] | Moderate |
| **Hamauchi 2019** | Serious[a] | Low | Low | Low | Low | Low | Low[c] | Serious |
| **Han 2023** | Moderate | Low | Low | Low | Low | Low | Low[c] | Moderate |
| **Imai 2022** | Moderate | Low | Low | Low | Low | Low | Low[c] | Moderate |
| **Magnes 2021** | Moderate | Low | Low | Low | Low | Low | Moderate[b] | Moderate |
| **Maring 2018** | Moderate | Low | Low | Low | Low | Low | Low[c] | Moderate |
| **Nassif 2022** | Moderate | Low | Low | Low | Low | Low | Low[c] | Moderate |

| Study | Bias due to confounding | Bias in selection of participants | Bias in classification of interventions | Bias due to deviations from intended interventions | Bias due to missing data | Bias in measurment of outcomes | Bias in selection of reported results | Overall risk of bias judgment |
|---|---|---|---|---|---|---|---|---|
| **Pryor 2009** | Moderate[c] | Low | Low | Low | Low | Low | Moderate[b] | Moderate |
| **Rades 2023** | Moderate | Low | Low | Low | Low | Low | Low[c] | Moderate |
| **Rambeau 2017** | Serious[a] | Low | Low | Low | Low | Low | Low[c] | Serious |
| **Saigal 2014** | Serious[a] | Low | Low | Low | Low | Low | Moderate[b] | Serious |
| **Srinivas 2020** | Serious[a] | Low | Low | Low | Low | Low | Moderate[b] | Serious |
| **Sun 2022** | Moderate | Low | Low | Low | Low | Low | Low[c] | Moderate |
| **Swiecicki 2020** | Moderate[c] | Low | Low | Low | Low | Low | Low[c] | Moderate |
| **Van Der Linden 2013** | Serious[a] | Low | Low | Low | Low | Low | Moderate[b] | Serious |
| **Ueki 2023** | Moderate[c] | Low | Low | Low | Low | Low | Low[c] | Moderate |
| **Weiss 2020** | Moderate[c] | Low | Low | Low | Low | Low | Low[c] | Moderate |
| **Ye 2013** | Moderate | Low | Low | Low | Low | Low | Low[c] | Moderate |

a: These studies did not perform any analysis adjusted for confounders; therefore, they were considered at serious risk of bias in the first domain; b: Retrospective studies usually lack a published protocol before the study starts. Thus, we considered studies lacking a detailed description of the methods section as 'moderate' risk of bias in this domain; c: Prospective studies or phase II trials were judged as moderate risk of bias regardless of analysis adjusted for confounders since fewer confounders are expected compared to retrospective studies.

**(B) Risk of bias summary for randomized studies (RoB 2).**

| Study | Bias from randomization process | Bias due to deviations from intended interventions | Bias due to missing outcome data | Bias in measurement of the outcomes | Bias in selection of the reported result | Overall risk of bias |
|---|---|---|---|---|---|---|
| **Patil 2023** | Low | Low | Low | Low | Low | Low |
| **Tao 2023** | Low | Low | Low | Low | Low | Low |
